# Supplementary material for: Immune response induced by the recombinant novel coronavirus vaccine (Adenovirus type 5 vector) (Ad5-nCoV) in persons living with HIV (PLWH)
Source: PLoS One. 2025 Jun 11;20(6):e0312893. doi: 10.1371/journal.pone.0312893 (PMC12157306; doi:10.1371/journal.pone.0312893)
Supplement: S1 protocol — (DOCX) [file pone.0312893.s003.docx]

Protocol FH-58 (AD5NCOV-HIV2b) Version 1.5 September 10^th^, 2021 **Clinical Trial Protocol**

**Study Sponsor:** Fundación Huésped

**Primary Study Product**

Recombinant Novel Coronavirus Vaccine (Adenovirus Type 5 Vector) (Ad5-nCoV)

**Date of protocol** September 10^th^, 2021

**Version** 1.5

**Title** Phase IIb Trial of Recombinant Novel Coronavirus Vaccine (Adenovirus Type 5 Vector) (Ad5-nCoV) in Adults 18 years of age and older, living

with HIV

**^Detailed Title^** A phase IIb clinical trial to evaluate the efficacy, safety and immunogenicity of two doses of Recombinant Novel Coronavirus

Vaccine (Adenovirus Type 5 Vector) in adults 18 years of age and older,

living with HIV.

**Protocol Number** FH-58 (AD5NCOV-HIV2b)

**Registration Number** TBD

Dr. Pedro Enrique Cahn, MD, PhD

**Principal**

**Investigator**

**Global Co-Principal**

Director, Fundación Huésped

Carlos Gianantonio 3932 (ex Pje. Ángel Peluffo) C1202ABB, Buenos Aires, Argentina

Telephone:(5411) 49817777

Fax: (5411) 49824024

E-mail: pedro.cahn@huesped.org.ar

Scott A Halperin, MD

Canadian Center for Vaccinology

Page **1** of **84**

Protocol FH-58 (AD5NCOV-HIV2b) Version 1.5 September 10^th^, 2021

**Investigator** Dalhousie University/IWK Health Centre

5850/5980 University Avenue

Halifax, NS B3K 6R8, Canada

Telephone: 902-470-8141

Fax: 902-470-7232

Email: scott.halperin@dal.ca

**Study Statisticians** Bruce Smith, PhD

Canadian Center for Vaccinology

Dalhousie University/IWK Health Centre

5850/5980 University Avenue

Halifax, NS B3K 6R8, Canada

Telephone: 902-470-8141

Fax: 902-470-7232

Email: bruce.smith@dal.ca

Lingyun Ye, MSc

Canadian Center for Vaccinology

Dalhousie University/IWK Health Centre

5850/5980 University Avenue

Halifax, NS B3K 6R8, Canada

Telephone: 902-470-8141

Fax: 902-470-7232

Email: Lingyun.Ye@iwk.nshealth.ca

**Study Sponsor**

**Scientific and Medical Affairs Contact**

**Study Financial Sponsor Contact**

Luis Barreto, MD

Senior Scientific Advisor and member of the Scientific Advisory Board CanSino Biologics, Inc.

185 South Avenue

TEDA West, Tianjin, China 300462

Telephone: 416-294-5840

Email: drluisbarreto@gmail.com

Tao Zhu, PhD

CanSino Biologics Inc.

185 South Avenue, TEDA West

Tianjin, China 300462

Tel:+86 13951994867

E-mail: tao.zhu@cansinotech.com

Page **2** of **84**

DocuSign Envelope ID: F44BE397-221A-4F0B-840B-52A8EAA64913 DocuSign Envelope ID: CD06F8BF-7BA1-4865-8AD6-CDF085D4307E

Protocol FH-58 (AD5NCOV-HIV2b) Version 1.5 September 10^th^, 2021

**Protocol Medical Writer**

Laura Leppan, BScN, RN

Canadian Center for Vaccinology Dalhousie University/IWK Health Centre 5850/5980 University Avenue. Email: leppanlaura@gmail.com

Page **3** of **84**

DocuSign Envelope ID: CD06F8BF-7BA1-4865-8AD6-CDF085D4307E

DocuSign Envelope ID: F44BE397-221A-4F0B-840B-52A8EAA64913

Protocol FH-58 (AD5NCOV-HIV2b) Version 1.5 September 10^th^, 2021

STATEMENT OF COMPLIANCE

**Signature Page of Sponsor’s Approval for the Clinical Trial Protocol**

Brief Title Phase IIb trial of Recombinant Novel Coronavirus Vaccine (Adenovirus Type 5 Vector) (Ad5-nCoV) in adults 18 years of age and older, living

with HIV

Official Title A phase IIb clinical trial to evaluate the efficacy, safety and immunogenicity of two doses of Recombinant Novel Coronavirus

Vaccine (Adenovirus Type 5 Vector) in adults 18 years of age and older.

Vaccine Recombinant Novel Coronavirus Vaccine (Adenovirus Type 5 Vector) (Ad5-nCoV)

Protocol Number FH-58 (AD5NCOV-HIV2b)

Protocol Date September 10^th^, 2021

Version No. 1.5

Sponsor Fundación Huésped

Sponsor Person In

Charge

Sponsor Person in Charge

Pedro Cahn, MD, PhD

Fundación Huésped

Carlos Gianantonio 3932, CABA, Argentina C1202ABB Telephone +54 9 11 49817777

Email: pcahn@hhuesped.org.ar

Signature: Date:

03-nov.-2022

Page **4** of **84**

DocuSign Envelope ID: CD06F8BF-7BA1-4865-8AD6-CDF085D4307E

DocuSign Envelope ID: F44BE397-221A-4F0B-840B-52A8EAA64913

Protocol FH-58 (AD5NCOV-HIV2b) Version 1.5 September 10^th^, 2021

**Statement of PI**

I agree to:

Take the full responsibilities as Global Principal Investigator (PI) of this clinical trial. Ensure that this clinical trial is conducted according to this approved protocol, or revised protocol, and the clinical trial SOPs from the sponsors.

Ensure that the investigators participating in this clinical trial understand the product information of the investigational vaccine provided by the Sponsors, and understand the duties and responsibilities related to the clinical trial as outlined in this clinical trial protocol.

Ensure that there are no changes to the clinical trial protocol without the review and written approval of the sponsors and the Institutional Review Board (IRB) unless it is due to urgent removal of immediate damages to the participants or due to the regulatory requirements (such as due to administration requirements).

That I fully understand the correct usage methods of the investigational vaccine, and I fully understand the information provided by the sponsors, including but not limited to the following: Current Investigator Brochure [10] or equivalent documents.

I am familiar with and will comply with the requirements of Good Clinical Practices (GCP) and other relevant regulatory requirements.

Brief Title Phase IIb trial of Recombinant Novel Coronavirus Vaccine (Adenovirus Type 5 Vector) (Ad5-nCoV) in adults 18 years of age and older, living with

HIV

Protocol Number ^FH-58 (AD5NCOV-HIV2b)^

Protocol Date September 10th, 2021

Version No. 1.5

Principal

_Investigator_ Dr. Pedro Enrique Cahn, MD, PhD

Signature: Date:

03-nov.-2022

Page **5** of **84**

DocuSign Envelope ID: F44BE397-221A-4F0B-840B-52A8EAA64913 DocuSign Envelope ID: CD06F8BF-7BA1-4865-8AD6-CDF085D4307E

Protocol FH-58 (AD5NCOV-HIV2b) Version 1.5 September 10^th^, 2021 Page **6** of **84**

DocuSign Envelope ID: F44BE397-221A-4F0B-840B-52A8EAA64913 DocuSign Envelope ID: CD06F8BF-7BA1-4865-8AD6-CDF085D4307E

Protocol FH-58 (AD5NCOV-HIV2b) Version 1.5 September 10^th^, 2021

**Statement of Global Co-PI**

I agree to:

Take the full responsibilities as Global Principal Investigator (PI) of this clinical trial. Ensure that this clinical trial is conducted according to this approved protocol, or revised protocol, and the clinical trial SOPs from the sponsors.

Ensure that the investigators participating in this clinical trial understand the product information of the investigational vaccine provided by the Sponsors, and understand the duties and responsibilities related to the clinical trial as outlined in this clinical trial protocol.

Ensure that there are no changes to the clinical trial protocol without the review and written approval of the sponsors and the Institutional Review Board (IRB) unless it is due to urgent removal of immediate damages to the participants or due to the regulatory requirements (such as due to administration requirements).

That I fully understand the correct usage methods of the investigational vaccine, and I fully understand the information provided by the sponsors, including but not limited to the following: Current Investigator Brochure [10] or equivalent documents.

I am familiar with and will comply with the requirements of Good Clinical Practices (GCP) and other relevant regulatory requirements.

Brief Title Phase IIb trial of Recombinant Novel Coronavirus Vaccine (Adenovirus Type 5 Vector) (Ad5-nCoV) in adults 18 years of age and older, living with

HIV

Protocol Number ^FH-58 (AD5NCOV-HIV2b)^

Protocol Date September 10^th^, 2021

Version No. 1.5

Principal

_Investigator_ Dr. Scott A Halperin, MD

Signature: Date:

10-Nov-2022

Page **7** of **84**

DocuSign Envelope ID: F44BE397-221A-4F0B-840B-52A8EAA64913 DocuSign Envelope ID: CD06F8BF-7BA1-4865-8AD6-CDF085D4307E

Protocol FH-58 (AD5NCOV-HIV2b) Version 1.5 September 10^th^, 2021

**STUDY SYNOPSIS**

| **Study Title** | Phase IIb trial of Recombinant Novel Coronavirus Vaccine (Adenovirus Type 5 Vector) (Ad5- nCoV) in adults 18 years of age and older, living with HIV. |
| --- | --- |
| **Full Title** | A phase IIb clinical trial to evaluate the efficacy, safety and immunogenicity of two doses of Recombinant Novel Coronavirus Vaccine (Adenovirus Type 5 Vector) in adults 18 years of age and older, living with HIV. |
| **Objectives** | **Primary Safety Objectives**:  ● Evaluate the incidence of solicited adverse reactions within 7 days after vaccination. ● Evaluate the incidence of unsolicited adverse events within 28 days after vaccination. ● Evaluate the HIV viral load 24 and 52 weeks after vaccination  ● Evaluate the incidence of serious adverse events (SAE) and medically attended adverse events (MAE) within 52 weeks after vaccination in all participants.  **Primary Immunogenicity Objectives**:  ● Evaluate the seroconversion rate of S-RBD IgG antibody on Day 28, Day 84 and Week 24 and Week 52 after vaccination, measured by ELISA.  ● Evaluate the immunogenicity of two doses of the vaccine.  **Secondary Safety Objectives:**  ● Evaluate the incidence of a decrease in CD4+ cell count by ≥20% at 24 and 52 weeks after vaccination.  ● Evaluate changes in the CD4/CD8 ratio at 24 and 52 weeks compared to the basal value. ● To evaluate the efficacy of two doses of Ad5-nCoV in different age groups from 14 and 28 days to 24 and 52 weeks after vaccination. This will be evaluated by weekly participant contact to assess for any signs or symptoms of COVID 19.  **Secondary Immunogenicity Objectives:**  ● Evaluate the GMT of S-RBD IgG antibody on Day 28, Day 84 and Week 24 and Week 52 after vaccination, measured by ELISA.  ● Evaluate the GMI of S-RBD IgG antibody on Day 28, Day 84 and Week 24 and Week 52 after vaccination, measured by ELISA.  ● Evaluate the seroconversion rate of pseudo-virus neutralizing antibody on Day 28, Day 84 and Week 24 and Week 52 after vaccination.  ● Evaluate the GMT of pseudo-virus neutralizing antibody on Day 28, Day 84 and Week 24 and Week 52 after vaccination.  ● Evaluate the GMI of pseudo-virus neutralizing antibody on Day 28, Day 84 and Week 24 and Week 52 after vaccination.  ● Evaluate the positive rate and level of IFN-γ, TNF, IL-4, IL-5, IL-13 stimulated by peptide |

Page **8** of **84**

DocuSign Envelope ID: F44BE397-221A-4F0B-840B-52A8EAA64913 DocuSign Envelope ID: CD06F8BF-7BA1-4865-8AD6-CDF085D4307E

Protocol FH-58 (AD5NCOV-HIV2b) Version 1.5 September 10^th^, 2021

|  | pool of S protein on Day 28, Day 84 and Weeks 24 and Week 52 after vaccination, measured by intracellular cytokine staining (ICS) (in a subset of approximately 50 participants).  **Exploratory Objectives**  ● To evaluate the efficacy of two doses of Ad5-nCoV in preventing virologically confirmed (PCR positive) COVID-19 disease occurring 14 days and 28 days to 52 weeks after vaccination, regardless of severity.  ● To evaluate the efficacy of two doses of Ad5-nCoV in preventing virologically (PCR) or serologically (four-fold increase in SARS-CoV-2 anti-N IgG from pre-immunization to post symptom, defined as Day 21-28 post illness blood test, or pre-symptom to post-symptom blood test) confirmed COVID-19 disease occurring 14 and 28 days to 52 weeks after vaccination, regardless of severity.  ● To evaluate the efficacy of two doses of Ad5-nCoV in preventing severe COVID-19 disease caused by SARS-CoV-2 infection from 14 and 28 days to 24 and 52 weeks after vaccination.  *Severe disease is defined as: 1) Clinical signs at rest indicative of severe systemic illness (respiratory rate ≥ 30 per minute, heart rate ≥ 125 per minute, SpO2 ≤ 93% on room air at sea level or PaO2/FiO2 < 300 mm Hg), 2) Respiratory failure (defined as needing high-flow oxygen, non-invasive ventilation, mechanical ventilation or ECMO), 3) Evidence of shock (SBP < 90 mm Hg, DBP < 60 mm Hg, or requiring vasopressors), 4) Significant acute renal, hepatic, or neurologic dysfunction, 5) Admission to an ICU*  ● Evaluate the efficacy of Ad5-nCoV in preventing asymptomatic disease of COVID-19 (confirmed by N IgG antibody on week 52 after vaccination).  ● Evaluate the severity of COVID-19 cases among vaccine recipients (based on WHO or FDA criteria) as compared to the control group, to measure antibody-mediated disease enhancement (ADE).  ● Evaluate for any evidence of SARS-CoV-2 virus shedding in COVID-19 cases that occurred 28 days to 52 weeks after vaccination (detection of viral nucleic acid every 2 days after being confirmed).  ● Perform genotyping of SARS-CoV-2 virus isolates of COVID-19 cases that occurred 28 days to 52 weeks after vaccination.  ● Evaluate incidence of suspected but unconfirmed cases of COVID-19 (either because of negative or no tests). |
| --- | --- |
| **Indication** | Prevention of novel coronavirus disease (COVID-19) caused by infection with the SARS-CoV-2 coronavirus. |
| **Target**  **population** | Generally healthy adults 18 years of age and older, living with HIV |

Page **9** of **84**

DocuSign Envelope ID: F44BE397-221A-4F0B-840B-52A8EAA64913 DocuSign Envelope ID: CD06F8BF-7BA1-4865-8AD6-CDF085D4307E

Protocol FH-58 (AD5NCOV-HIV2b) Version 1.5 September 10^th^, 2021

| **Sample Size** | N=500 |
| --- | --- |
| **Rationale**  **for study** | The 2019 novel coronavirus (SARS-CoV-2) is a positive non-segment single-stranded RNA virus, belonging to the Coronaviridae family of Nidovirales. There are six known coronaviruses in humans, including 229E and NL63 in the alpha genus, OC43 and HKU1 in the beta genus, Middle East respiratory syndrome-associated coronavirus (MERS-CoV), and severe acute respiratory syndrome-associated coronavirus (SARS- CoV).  The coronavirus isolated from the lower respiratory tract of patients with unexplained pneumonia in Wuhan is a new type of genus β coronavirus. Following the outbreak of severe acute respiratory syndrome coronavirus (SARS-CoV) in 2002 and the outbreak of MERS-CoV in 2012, SARS-CoV 2 is the third highly pathogenic coronavirus. The Coronavirus Disease 2019 (COVID-19) is caused by the infection of the 2019 novel coronavirus (SARS-CoV-2) started in January 2020. World Health Organization (WHO) declared COVID-19 a worldwide pandemic on March 12, 2020. As of Oct.20, 2020, there were over 42 million cases worldwide and over 1 million deaths in 185 countries/regions [1]. China had reported 87,028 confirmed cases, the US has over 8 million cases, and Canada over 200,000 cases [1]. As of November 28, 2020, there were over 61.8 million cases worldwide and over 1.4 million deaths in 185 countries/regions [1]. The COVID-19 pandemic has brought heavy economic pressure, medical burden, and severe harm to people's lives and health.  It is necessary and very urgent to develop preventive vaccines against COVID-19 to meet the global needs.  The vaccine candidate Ad5-nCoV, intended to prevent the COVID-19 caused by SARS-CoV-2, is jointly developed by CanSino Biologics Inc. and its collaborator, the Beijing Institute of Biotechnology. Using the replication-deficient human adenovirus type 5 as a vector, Ad5-nCoV has been developed through recombinant virus construction, amplification, cell culture, purification, and formulation. Ad5-nCoV expresses the specific S protein of SARS-CoV-2. It is suggested that both humoral and cellular immune responses play an important role in protective immunity according to the results of pre-clinical, phase I, and phase II studies conducted by CanSino Biologics. This study is a phase IIb clinical trial to evaluate the efficacy, safety, immunogenicity of two doses of Ad5-nCoV at a dose level of 5×10^10^ vp in healthy adults, aged 18 years of age and older living with HIV. |
| **Testing**  **Vaccine** | **Study Product:** Ad5-nCoV manufactured by CanSino Biologics Inc.  **Active Ingredients:** Replication-defective recombinant human type 5 adenovirus expressing S protein of novel coronavirus  **Excipients:** Mannitol, sucrose, sodium chloride, magnesium chloride, polysorbate 80, HEPES, and glycerin  **Packaging**: The vaccine is contained in a vial  **Specification:** 0.5 mL/vial  **Dosage**: 5x10^10^vp (≥4x10^10^vp) |

Page **10** of **84**

DocuSign Envelope ID: F44BE397-221A-4F0B-840B-52A8EAA64913 DocuSign Envelope ID: CD06F8BF-7BA1-4865-8AD6-CDF085D4307E

Protocol FH-58 (AD5NCOV-HIV2b) Version 1.5 September 10^th^, 2021

|  | **Shelf Life**: Tentatively 24 months  **Storage:** It is recommended to store and transport at 2-8 ℃ (details of the temperature requirements are specified in the pharmacy manual).  **Administration:** Intramuscular (IM) injection in the deltoid muscle of the upper arm. **Schedule:** A double-dose of vaccine is scheduled for all participants. A second dose of vaccine is scheduled for all participants at Day 56.  **Developers:** CanSino Biologics Inc.  Beijing Institute of Biotechnology  **Manufacturer**: CanSino Biologics Inc. |
| --- | --- |
| **Study design** | **Summary:**  This Phase IIb study is a clinical trial for 500 participants 18 years of age or older living with HIV. All participants will receive two doses of the study vaccine and will be followed to monitor vaccine candidate safety, immunogenicity, and efficacy during 52 weeks. Fifty five days after the first vaccination, all participants will receive a second injection.  **Study Interventions:**  **All participants will be subject to the following procedure**s:  **● Informed consent**  The signed informed consent must be obtained before study participation.  **● Check inclusion and exclusion criteria**  All inclusion and exclusion criteria will be checked at the screening in-person visit. **● Collect demographic data and participant contact information**.  Record participant’s demographic data such as date of birth, sex, gender, height, weight and race in the participant’s eCRF. A current email addresses and or phone numbers will be collected for each participant. Contact information may also be used to remind participants of up-coming in-person visits.  **● Medical history**  Obtain the participant’s medical history by interview and/or review of the participant’s medical records and record any pre-existing conditions or signs and/or symptoms present in a participant prior to the first study injection in the eCRF. This will include reviewing any health condition that may prevent the participant from enrolling in the study, such as an unstable health condition. HIV clinical, treatment, and laboratory history will be reviewed. **● HIV status, viral load, CD4 and CD8 counts**  **●** At screening visit, HIV status will be verified based on a previous serologic result or previous detectable viral load. HIV status can also be confirmed with a prescription for antiretroviral treatment or a follow-up record from your treating physician. Viral load, and CD4 and CD8 counts samples will be collected at the first screening/baseline visit, randomization visit, and week 24 and week 52 visits. Screening visit value will be used as reference value for |

Page **11** of **84**

DocuSign Envelope ID: F44BE397-221A-4F0B-840B-52A8EAA64913 DocuSign Envelope ID: CD06F8BF-7BA1-4865-8AD6-CDF085D4307E

Protocol FH-58 (AD5NCOV-HIV2b) Version 1.5 September 10^th^, 2021

|  | comparison with further values.  **● Check contraindications, warnings and precautions to injection**  Contraindications, warnings and precautions to injection must be checked at the beginning of the First Injection Visit.  **● Urine Pregnancy Test/Birth Control**  Women of child-bearing age will be asked to perform a urine pregnancy test before the Injection Visits. The result of the urine pregnancy test must be negative in order to proceed with vaccine administration. In addition, participants who are able to become pregnant or could impregnate a partner are required to have used approved contraception for least 30 days prior to the study vaccination, and commit to use it for 90 days after the study vaccination. **● Screening conclusion**  Participants will be deemed eligible to participate upon reviewing medical history and inclusion and exclusion criteria. This will occur prior to vaccination on First Injection Visit and will be verified before Second Injection.  **● Assess pre-injection vital signs (body temperature, blood pressure, pulse rate and respiratory rate)**  The body temperature of all participants needs to be measured prior to any study product administration on First and Second Injection Visits. Body temperature may be measured by any method (oral, axillary). If the participant has fever (fever is defined as temperature ≥38.0° C orally or ≥37.8 axillary) on the day of injection, the injection will be rescheduled within 1 week.  **● Check and record prior medications and concomitant medication/injection** Prior medications and concomitant medication/injection must be checked and recorded in the eCRF. Prior medications should include any medication taken by the participant within 14 days prior to screening. Participants will be asked to avoid over-the-counter medications such as antipyretics (e.g., acetaminophen) and anti-inflammatory medications (e.g., ibuprofen, naproxen) in the 12 hours before study vaccine receipt but will be allowed to take these over the-counter medications as needed to treat fever or other AE after vaccination. Usage of these over-the-counter medications will be recorded as concomitant medications and linked to the AE collected as solicited or unsolicited events, as the case may be.  **● Check and record intercurrent medical conditions**  Any medical conditions should be collected in the clinical chart and recorded in the eCRF. **● Baseline serology**  Approximately 25 mL of whole blood will be collected from each participant at First Injection Visit, Day 28, Day 84, week 24 and week 52 and separated for serum. The serum will be aliquoted. The results of antibody testing from baseline serum will not be used to determine eligibility for enrolment; instead, results will solely be used for analysis and comparison purposes. Presence of pre-existing SARS-CoV-2 antibodies found in baseline serum will be used for a stratified immunogenicity and efficacy sub-analysis. The second aliquot will be stored as a back-up. For participants who entered in the blinded version and then were |
| --- | --- |

Page **12** of **84**

DocuSign Envelope ID: F44BE397-221A-4F0B-840B-52A8EAA64913 DocuSign Envelope ID: CD06F8BF-7BA1-4865-8AD6-CDF085D4307E

Protocol FH-58 (AD5NCOV-HIV2b) Version 1.5 September 10^th^, 2021

|  | unblinded and vaccinated, baseline serology will not be performed again, taking the values from the baseline visit performed at the first visit.  **●** For linking purposes, each participant will be assigned a treatment identification number. The number of each administered treatments must be recorded in the eCRF.  **● Injection of study vaccine**  After completing all prerequisite procedures prior to injection, one dose of the assigned vaccine will be administered IM in the deltoid muscle, preferable of the non-dominant arm. If the investigator or delegate determines that the participant’s health on the day of administration temporarily precludes administration, the visit will be rescheduled within 1 week. There will be a 15-minute wait after each vaccination to monitor for any rare anaphylactic reaction. At Day 56 after first vaccination, all participants will get a second injection of the investigational vaccine (Second Injection Visit)  **● Safety participant contacts**  Every 4 Weeks for 52 weeks there will be telephone calls following vaccination. They will be used to continuously collect any SAE and MAE data. Participants can report any AE they experience at any time during the study period; however, only SAE and MAE will be entered into the eCRF. In-person visits will occur for Day 28 visit (to collect solicited and unsolicited events, Day 56 (Second Injection Visit), Day 84 and Week 24, and Week 52 visits. **● Efficacy participant contacts**  All participants will have efficacy participant contacts weekly, for 52 weeks following vaccination. There will be approximately 52 efficacy participant contacts. All efficacy participant contacts will be emails, although other methods such as phone calls or text messages, if preferred, are acceptable. Each participant contact needs to be recorded in the study  electronic case report form. The purpose of these participant contacts is to ask if the participants had any symptoms of illness they may be experiencing, regardless of severity. Any possible COVID-19 relevant symptoms will trigger laboratory testing for SARS-CoV-2 infection.  **● Illness participant contact visits (Confirmed or suspected infection during study)** Participants will be provided detailed instructions regarding the signs and symptoms of COVID-19 disease at each participant contact and will be instructed to seek medical attention and notify study staff should symptoms occur. Symptoms of interest include fever, cough, difficulty breathing, diarrhea, nausea, vomiting, prolonged fatigue, respiratory symptoms, chills, myalgia, sore throat, headache, congested/runny nose, pneumonia, difficulty swallowing, anosmia/ageusia (loss of sense of smell/taste), and neurological events.  During the observation period of the study, if any of these symptoms develop in a participant, he/she should immediately follow local procedures for care of suspected COVID-19 illness and contact the study team. If a COVID-19 infection is found during the study, a case investigation will be undertaken according to locally recommended procedures.  **●** If COVID-19 is suspected, the participant will have a nasopharyngeal/throat swab taken. |
| --- | --- |

Page **13** of **84**

DocuSign Envelope ID: F44BE397-221A-4F0B-840B-52A8EAA64913 DocuSign Envelope ID: CD06F8BF-7BA1-4865-8AD6-CDF085D4307E

Protocol FH-58 (AD5NCOV-HIV2b) Version 1.5 September 10^th^, 2021

|  | Testing for COVID-19 by PCR will be performed at the local clinical laboratory. **●** If negative, the test will be repeated in 3 days from the first testing day.  **●** All attempts will be made to re-test positive samples in a reference laboratory. If the local lab is also a reference laboratory, then a confirmatory sample is not required.  **●** If the PCR is positive, nasopharyngeal/throat swabs will be taken every 2 days times 3 and then weekly and tested for COVID-19 by PCR until a negative sample is obtained. **●** A serum sample (10mL blood) will be collected at the time of illness presentation and testing for SARS-CoV-2 anti-N IgG antibodies will be performed at Nexelis Laboratories (Canada). **●** At Day 21-28 post-illness, a second serum sample will be taken and sent to the central laboratory for anti-N IgG.  **●** Participants who were PCR negative will also have that visit on day 21-28, where they will be tested for SARS-CoV-2 antibodies (Nexelis Laboratories (Canada)). This visit takes place at the research site where the participant is enrolled.  **●** Participants who develop COVID-19 infection post-vaccination will be carefully monitored for vaccine-related disease enhancement in collaboration with their primary physician who will be encouraged to obtain specimens in accordance with the Brighton Collaboration definition (Munoz, 2020), including a serum sample tested locally for C-reactive protein (CRP), ferritin, and procalcitonin, biomarkers that have been associated with predicting more severe disease outcome in infected patients.  Participants who develop COVID-19 disease will continue to be followed for other study outcomes by telephone while in isolation, and at the study site once recovered and released from isolation by public health officials.  **● Final serology**  Approximately 25mL of whole blood will be collected and will then be separated into serum at week 52. The serum will be aliquoted. The first aliquot will measure anti-N IgG antibodies, at the Nexelis Laboratories (Canada). The second aliquot will be stored as a back-up. The one year antibody levels measured against anti-N IgG antibodies will be compared to the participant’s baseline levels to evaluate the exploratory efficacy analysis against asymptomatic infection.  **● E-diary and Diary Cards**  Participants will be provided with access to an e-diary and a thermometer, and instructions about how to use them. A paper diary card may be available if required by the study site. Participants will be asked to record solicited AE for 7 days after receiving the vaccination, and unsolicited AE for 28 days in their diary. A second e-diary or a paper diary will be provided at Day 56 and will be collected at Day 84 visit. The completed e-diary and diary cards will be reviewed and data collected at the 28-Day safety in-person visit. Non completion of the e-diary or diary cards will be investigated with the participant through telephone call(s) or any other convenient procedure. All data collected from the e-diary and diary cards will be uploaded into |
| --- | --- |

Page **14** of **84**

DocuSign Envelope ID: F44BE397-221A-4F0B-840B-52A8EAA64913 DocuSign Envelope ID: CD06F8BF-7BA1-4865-8AD6-CDF085D4307E

Protocol FH-58 (AD5NCOV-HIV2b) Version 1.5 September 10^th^, 2021

|  | the eCRF.  **● Immunogenicity in-person visit**  **1. Blood collection for ELISA and pseudo-virus neutralization antibody measurement** Approximately 25mL of whole blood will be collected on First Injection Visit, Day 28, Day 84, Week 24, and Week 52, as described above. These in-person visits will occur at the participant’s local study site. Serum will be separated into two aliquots. A baseline antibody titer against SARS-CoV-2 will be established from the blood sample collected on First Injection Visit, as outlined in the “baseline serology” description. In addition, these samples will also be used to evaluate for additional immunogenicity objectives including: the seroconversion rate of S-RBD IgG antibody, GMT of S-RBD IgG antibody, GMI of S-RBD IgG antibody,  seroconversion rate of pseudo-virus neutralizing antibody, GMT of pseudo-virus neutralizing antibody, and the GMI of pseudo-virus neutralizing antibody at Day 28, Day 84, Week 24, and Week 52. The second aliquot will be stored as a back-up. **2. Blood collection for ICS measurement**  In a subset of approximately 50 participants, approximately 30mL of whole blood will be collected. Collections will occur on First Injection Visit (baseline), just prior to vaccination, and on Day 28, Day 84, and at 24 and 52 weeks post-vaccination. TNF, IL-4, IL-5, IL-13 will be measured by ICS on Day 28, Day 84 and Weeks 24 and 52 after vaccination. PBMC will be shipped on liquid nitrogen to Nexelis Laboratories (Canada).  **Study duration:**  Total duration of this trial from the enrolment to the last visit will be approximately one year (52 weeks). |
| --- | --- |
| **Visit plan** | All participants will have at least 6 (six) planned in-person visits on Screening visit/First Injection Visit and Day 28, Day 56 (Second Injection Visit), Day 84, Week 24, and Week 52. The Screening/baseline visit will entail reviewing the ICF, completing a medical history of the participant, reviewing inclusion and exclusion criteria, and collecting a blood sample for HIV viral load and CD4/CD8 counts. A baseline SARS-CoV-19 antibody (Nexelis Laboratories), and a urine pregnancy test in women of child-bearing potential will be performed. The vaccine will be administered after having the negative result on the pregnancy test. Participants are required to remain at the study site for a minimum of 15 minutes after receiving the vaccine to monitor for any vaccine-related AE.  Participants will be given access to an e-diary or paper diary card to record solicited AE from First Injection Visit- day 7 and unsolicited AE from First Injection Visit- day 28. The data in the completed e-diary or diary card will be reviewed and data will be collected on Day 28.  A new e-diary or paper diary card to record solicited and unsolicited AE from Second Injection will be provided at Second Injection Visit- Day 56. The data in the completed e-diary or diary card |

Page **15** of **84**

DocuSign Envelope ID: F44BE397-221A-4F0B-840B-52A8EAA64913 DocuSign Envelope ID: CD06F8BF-7BA1-4865-8AD6-CDF085D4307E

Protocol FH-58 (AD5NCOV-HIV2b) Version 1.5 September 10^th^, 2021

|  | provided during the Day-56 visit will be reviewed and data will be collected at visit on Day 84. Further details are outlined in Section 3.7 and 5.1.  All participants will be contacted on a weekly basis to be reminded to report any signs or symptoms of illness to study staff. Participants will either be contacted by the site weekly, or they will receive an automated message (email) with a link to answer questions about whether or not they had an SAE, an MAE or COVID-19 symptoms. Participants will also be asked on their day 7 contact about any events that might meet a pause or early termination criterion. The reporting and monitoring of any participant illness are consistent with meeting the primary efficacy objective. If a participant becomes ill, they will be asked to immediately proceed to the site designated by their local study team where they will be assessed and treated as deemed appropriate. Participants will also receive a telephone call every 4 weeks, to evaluate for the incidence of any SAE or MAE.  A subset of 50 participants will have additional immunologic testing performed, following the same visit schedule, to evaluate T-cell response to the Ad5-nCoV vaccine.  *If a participant demonstrates signs or symptoms of illness, an unplanned in-person visit may occur for evaluation and possible treatment. If, for any reason following an electronic participant contact, the investigator would like to conduct an in-person visit (at home or at the site) with a participant, they may do so at their discretion*. |
| --- | --- |
| **Pause and**  **early**  **termination** | **Study Suspension Criteria**  If a suspension criterion is met, the study will be put on hold, and further injections will not occur until a safety review has been conducted. Should a suspension criterion be met, the local PI will inform the PIs, Sponsors, and NRA within 24 hours.  **Suspension criteria includes:**  Grade 3 or greater AE occurring in >15% of participants will constitute study suspension criteria. To be included as suspension criteria, AE must begin within three days after study injection (day of injection and 2 subsequent days) and persist at Grade ≥ 3 on three consecutive days, depending upon symptom severity and kinetics.  A suspected, unexpected serious adverse reaction (SUSAR) that is life-threatening or results in death will warrant study suspension.  Other study suspension criteria include if the IDMC assessed the potential safety risks to be harmful, or if the vaccine candidate might be ineffective.  **Study Early Termination Criteria**  ● Required by the sponsor, or  ● Required by the regulatory authority, or  ● Required by an institutional review board (IRB) |

Page **16** of **84**

DocuSign Envelope ID: F44BE397-221A-4F0B-840B-52A8EAA64913 DocuSign Envelope ID: CD06F8BF-7BA1-4865-8AD6-CDF085D4307E

Protocol FH-58 (AD5NCOV-HIV2b) Version 1.5 September 10^th^, 2021

|  | **Process of Suspension of Injection and/or Study Modification**  In the event that a safety signal is observed, the IDMC might recommend that the sponsor suspend injections of all group participants (early termination of study) or selected groups. In this case, for impacted groups:  Participants who are already injected will continue all visits as planned.  Participants who signed an informed consent but have not received any study product will be informed that their study participation will be stopped. |
| --- | --- |
| **Statistical**  **analysis** | **Statistical Analysis Plan (SAP)**  **Analysis of Demographic and Baseline Characteristics**  Demographic and baseline characteristics will be summarized overall and then by treatment group using appropriate descriptive statistics. Continuous data will be summarized using the number of observations, mean, standard deviation, median, minimum and maximum. Categorical data will be summarized using frequency counts and percentages. 95% confidence intervals will be reported, but no statistical hypothesis testing will be conducted. Results will be presented for all participants.  **Analysis of Efficacy**  Due to limited sample size, no formal efficacy hypothesis will be tested as primary objective. A listing of baseline characteristics, treatment group and outcome for virologically confirmed (PCR positive) COVID-19 cases will be produced. An exploratory efficacy analysis of Ad5-nCoV in preventing asymptomatic disease of COVID-19 will be conducted at the end of the study.  **Safety Analysis**  **Primary Safety Endpoint:**  ● The incidence of solicited adverse reactions within 7 days after vaccination. ● The CD4 / CD8 ratio delta compared to baseline.  ● The incidence of unsolicited adverse events within 28 days after each vaccination. ● The incidence of detectable HIV viral load 24 and 52 weeks after vaccination ● The incidence of serious adverse events (SAE) and medically attended adverse events (MAE) within 52 weeks after vaccination in all participants.  Data from all participants will be used to assess the primary safety endpoint.  **Secondary Safety Objective Endpoints**  ● The incidence of a decrease in CD4+ cell count by ≥20% at 24 and 52 weeks after vaccination  All SAEs and MAEs will be listed and summarized by groups. |

Page **17** of **84**

DocuSign Envelope ID: F44BE397-221A-4F0B-840B-52A8EAA64913 DocuSign Envelope ID: CD06F8BF-7BA1-4865-8AD6-CDF085D4307E

Protocol FH-58 (AD5NCOV-HIV2b) Version 1.5 September 10^th^, 2021

|  | For each participant, the individual local events will be aggregated into a combined event “Local”, which is the maximum severity of the individual local events. For each participant, the individual general events will be aggregated into a combined event “General”, which is the maximum severity of the individual general events.  An aggregate event “Any” will be defined as the maximal severity of the combined events “Local” and “General”. In addition to being graded for severity as mild, moderate or severe, the severity of all events will be graded as "Any", which will include mild, moderate or severe events, and "Significant" which will include moderate or severe events.  All statistical tests on safety performed will be 2-sided with Type I error of 5%.  Missing values will not be included in the safety analyses, and there will be no imputation of missing values. No adjustments will be made for multiple comparisons.  For the analysis of proportions, binomial point estimates and exact binomial confidence intervals will be calculated.  Fisher’s Exact Test will be used to assess differences in rates of adverse events between treatment and control groups.  In addition, all safety data will be analysed descriptively, including unsolicited events collected through the end of the observation period.  **Immunogenicity Analysis**  **Primary Immunogenicity Endpoints**:  ● The seroconversion rate of S-RBD IgG antibody on Day 28, Day 84 and Week 24 and Week 52 after vaccination, measured by ELISA.  ● Evaluate the immunogenicity of one versus two doses of the vaccine  **Secondary Immunogenicity Endpoints**:  ● GMT of S-RBD IgG antibody on Day 28, Day 84 and Week 24 and Week 52 after vaccination, measured by ELISA.  ● GMI of S-RBD IgG antibody on Day 28, Day 84 and Week 24 and Week 52 after vaccination, measured by ELISA.  ● Seroconversion rate of pseudo-virus neutralizing antibody on Day 28, Day 84 Week 24 and Week 52 after vaccination.  ● GMT of pseudo-virus neutralizing antibody on Day 28, Day 84 and Week 24 and Week 52 after vaccination. |
| --- | --- |

Page **18** of **84**

DocuSign Envelope ID: F44BE397-221A-4F0B-840B-52A8EAA64913 DocuSign Envelope ID: CD06F8BF-7BA1-4865-8AD6-CDF085D4307E

Protocol FH-58 (AD5NCOV-HIV2b) Version 1.5 September 10^th^, 2021

|  | ● GMI of pseudo-virus neutralizing antibody on Day 28, Day 84 and Week 24 and Week 52 after vaccination.  ● Positive rate and level of IFN-γ stimulated by peptide pool of S protein on Day 28, Day 84 and Weeks 24 and Week 52 after vaccination, measured by ELISpot.  ● Positive rate and level of IL-2, IL-4, IL-13, and IFN-γ stimulated by peptide pool of S protein on days 28, Day 84 and Week 24 and Week 52 after vaccination, measured by intracellular cytokine staining (ICS).  Geometric mean antibody titers (GMTs) and GMI’s and their two side 95% confidence intervals will be calculated by group. Analyses will be performed on the logarithmically (base 10) transformed values. Individual titers below the detection limit will be set to half the limit.  For the analysis of proportions, binomial point estimates and exact binomial confidence intervals will be calculated for each group. Rates will be compared between groups using Fisher’s exact tests, and geometric means will be compared using t-tests.  All statistical tests performed for immunogenicity will be 2-sided with Type I error rate of 5%. Missing values will not be included in the immunogenicity analyses, and there will be no imputation of missing values. No adjustments will be made for multiple comparisons. The immunogenicity analysis will be carried out on both intention-to-treat and per-protocol cohorts.  **Exploratory Objective Endpoints**  ● Efficacy of two doses of Ad5-nCoV in preventing symptomatic disease of COVID-19 (confirmed by PCR) occurring 14 and 28 days to 52 weeks after vaccination, regardless of severity.  ● Efficacy of two doses of Ad5-nCoV in preventing virologically (PCR) or serologically (four-fold increase in SARS-CoV-2 anti-N IgG from pre-immunization to post symptom, defined as Day 21-28 post illness blood test, or pre-symptom to post-symptom blood test) confirmed COVID-19 disease occurring 14 and 28 days to 52 weeks after vaccination, regardless of severity.  ● Efficacy of Ad5-nCoV in preventing severe COVID-19 disease caused by SARS-CoV-2 infection from 14 and 28 days to 24 and 52 weeks after vaccination.  ● Efficacy of Ad5-nCoV in preventing asymptomatic disease of COVID-19 (confirmed by N IgG antibody on week 52 after vaccination).  ● Severity of COVID-19 cases among vaccine recipients (based on WHO or FDA criteria) as compared to the control group, to measure antibody-mediated disease enhancement (ADE). ● SARS-CoV-2 virus shedding in COVID-19 cases that occurred 28 days to 52 weeks after vaccination (detection of viral nucleic acid every 2 days after being confirmed). ● Genotype of SARS-CoV-2 virus isolates of COVID-19 cases that occurred 28 days to 52 weeks after vaccination. |
| --- | --- |

Page **19** of **84**

DocuSign Envelope ID: F44BE397-221A-4F0B-840B-52A8EAA64913 DocuSign Envelope ID: CD06F8BF-7BA1-4865-8AD6-CDF085D4307E

Protocol FH-58 (AD5NCOV-HIV2b) Version 1.5 September 10^th^, 2021

|  | ● Suspected but unconfirmed cases of COVID-19 (either because of negative or no tests) ● Evaluate incidence of suspected but unconfirmed cases of COVID-19 (either because of negative or no tests).  **Analysis of exploratory endpoints**  Analysis of exploratory endpoints will be descriptive. PCR confirmed positive subjects will be classified as having severe or non-severe disease. Severity of disease, SARS-CoV-2 virus shedding and Genotypes SARS-CoV-2 virus isolates will be listed by subject and date, along with demographic data, including the study centre. Suspected but unconfirmed cases and asymptomatic cases will also be listed.  **Final Analysis**  Immunogenicity analyses will be carried out using according-to-protocol cohorts in addition to intention-to-treat cohorts. |
| --- | --- |
| **Inclusion**  **criteria** | **Inclusion Criteria**  **●** Adults of 18 years of age, and older.  **●** Confirmed HIV infection defined by: A positive rapid test or ELISA and/or a plasma HIV-1 viral load >1000 copies/ml or rapid test or ELISA plus a positive Western Blot test. In patients on antiretroviral treatment, a medical history summary or antiretroviral prescription signed and stamped by the treating physician is acceptable.  **●** Able and willing (in the Investigator’s opinion) to comply with all study requirements. **●** Willing to allow the investigators to discuss the volunteer’s medical history with their General Practitioner/personal doctor and access all medical records when relevant to study procedures.  **●** Healthy adults, or stable-healthy adults who may have a pre-existing medical condition that does not meet any exclusion criteria. A stable medical condition is defined as disease not requiring significant change in therapy or hospitalization for worsening disease during the 3 months before enrolment [4].  **●** For subjects of childbearing potential only: Willingness to practice continuous effective contraception (see glossary) for 30 days prior to enrolment in the study, for 90 days after receiving vaccination during the study, and have a negative pregnancy test on the day(s) of vaccinations.  **●** Males participating in this study who are involved in heterosexual sexual activity must agree to practice adequate contraception (see glossary) and refrain from donating sperm for 90 days after receiving the study vaccination.  **●** Agreement to refrain from blood donation during the study.  **●** Provide written informed consent. |
| **Exclusion** | **Exclusion Criteria** |

Page **20** of **84**

DocuSign Envelope ID: F44BE397-221A-4F0B-840B-52A8EAA64913 DocuSign Envelope ID: CD06F8BF-7BA1-4865-8AD6-CDF085D4307E

Protocol FH-58 (AD5NCOV-HIV2b) Version 1.5 September 10^th^, 2021

| **criteria** | ● Participation in any other COVID-19 prophylactic drug trials for the duration of the study. o Note: Participation in COVID-19 treatment trials is allowed in the event of hospitalization due to COVID-19. The study team should be informed as soon as possible.  ● Planned receipt of any vaccine (licensed or investigational), other than the study intervention, within 14 days before and after study vaccination.  ● Prior receipt of an investigational or licensed vaccine likely to impact on the interpretation of the trial data (e.g. Adenovirus vectored vaccines, any coronavirus or SARS vaccines) ● Administration of immunoglobulins and/or any blood products within the three months prior to the planned administration of the vaccine candidate.  ● Any confirmed or suspected immunosuppressive or immunodeficient state; asplenia; recurrent severe infections and chronic use (more than 14 days) of immunosuppressant medication within the past 6 months. Topical steroids or short-term (course lasting ≤14 days) oral steroids are not an exclusion.  ● Active opportunistic infections or other AIDS-defining illness in the last six months. ● History of allergic disease or reactions likely to be exacerbated by any component of Ad5- nCoV  ● Any history of angioedema  ● Any history of anaphylaxis to any vaccine component  ● Pregnancy, lactation or willingness/intention to become pregnant within 90 days after receiving study vaccine  ● Current diagnosis of or treatment for cancer (except basal cell carcinoma of the skin and cervical carcinoma in situ)  ● History of serious psychiatric condition likely to affect participation in the study ● Bleeding disorder (e.g. factor deficiency, coagulopathy or platelet disorder), or prior history of significant bleeding or bruising following IM injections or venepuncture  ● Suspected or known current alcohol or drug dependency  ● Severe and/or uncontrolled cardiovascular disease, respiratory disease, gastrointestinal disease, liver disease, renal disease, endocrine disorder and neurological illness (mild/moderate well controlled comorbidities are allowed)  ● History of laboratory-confirmed COVID-19  ● Continuous use of anticoagulants, such as coumarins and related anticoagulants (i.e. warfarin) or novel oral anticoagulants (i.e. apixaban, rivaroxaban, dabigatran and edoxaban) ● Any other significant disease, disorder or finding which, in the opinion of the investigator, may significantly increase the risk to the volunteer because of participation in the study, affect the ability of the volunteer to participate in the study or impair interpretation of the study data. |
| --- | --- |

Page **21** of **84**

DocuSign Envelope ID: F44BE397-221A-4F0B-840B-52A8EAA64913 DocuSign Envelope ID: CD06F8BF-7BA1-4865-8AD6-CDF085D4307E

Protocol FH-58 (AD5NCOV-HIV2b) Version 1.5 September 10^th^, 2021 LIST OF KEY ROLES

**Key Roles/Duty Responsible Individual Organization**

Sponsor Pedro Cahn Fundación Huésped.

Carlos Gianantonio 3932,

CABA, Argentina

C1202ABB

Telephone +54 9 11 49817777

Email: pedro.cahn@huesped.org.ar

Principal Investigator Dr. Pedro Enrique Cahn, MD; PhD

Director, Fundación Huesped Fundación Huésped.

Carlos Gianantonio 3932, CABA, Argentina

C1202ABB

Telephone +54 9 11 49817777 Email: pcahn@huesped.org.ar

Global Co-PI Scott A Halperin Canadian Center for Vaccinology Dalhousie University/IWK Health Centre

5850/5980 University Avenue

Halifax, NS B3K 6R8, Canada

Tel: 902-470-8141

Email:scott.halperin@dal.ca

Central Safety Physician María Inés Figueroa Fundación Huésped. Carlos Gianantonio3932,

CABA, Argentina

C1202ABB

Telephone +54 9 11 49817777

Email:maria.figueroa@huesped.org.ar

Central laboratory Luc Gagnon Nexelis

525 Cartier West Blvd

Laval QC H7V 3S8

Canada

Tel: 450-239-1605

Page **22** of **84**

DocuSign Envelope ID: F44BE397-221A-4F0B-840B-52A8EAA64913 DocuSign Envelope ID: CD06F8BF-7BA1-4865-8AD6-CDF085D4307E

Protocol FH-58 (AD5NCOV-HIV2b) Version 1.5 September 10^th^, 2021 Email: luc.gagnon@nexelis.com

Central Data Manager Donna MacKinnon Cameron

Canadian Center for Vaccinology Dalhousie University/IWK Health Centre 5850/5980 University Avenue

Halifax, NS B3K 6R8, Canada

Tel: 902-470-8141

Email: donna.mackinnon

cameron@dal.ca

Central Statistician Bruce Smith Canadian Center for Vaccinology Dalhousie University/IWK Health Centre

5850/5980 University Avenue

Halifax, NS B3K 6R8, Canada

Telephone: 902-470-8141

Email: bruce.smith@dal.ca

Page **23** of **84**

DocuSign Envelope ID: F44BE397-221A-4F0B-840B-52A8EAA64913 DocuSign Envelope ID: CD06F8BF-7BA1-4865-8AD6-CDF085D4307E

Protocol FH-58 (AD5NCOV-HIV2b) Version 1.5 September 10^th^, 2021

***Table of Contents***

**Signature Page of Sponsor’s Approval for the Clinical Trial Protocol 4 Statement of PI 5 Statement of Global Co-PI 7**

***STUDY SYNOPSIS 8 GLOSSARY OF TERMS 30 1. INTRODUCTION 33***

**1.1. Background 33 1.2. Investigational vaccine 33 1.3. Rationale for the study 35**

***2. STUDY OBJECTIVES 37* 2.1 Primary Objectives 37 2.2 Secondary Objectives 37**

***3. STUDY DESIGN 39* 3.1 Summary 39 3.2 Study Groups 39 3.3 Sampling/Study Schedule 40 3.4 Procedures 41 3.5 Efficacy participant contacts 45 3.6 Immunogenicity In-Person Visits 47**

***4. STUDY POPULATION 47* 4.1 Inclusion Criteria 48 4.2 Exclusion Criteria 48 4.3 Recruitment 50**

***5. STUDY PROCEDURES AND INTERVENTIONS 50* 5.1 Study Intervention Descriptions 50 5.2 Study conclusion 55**

Page **24** of **84**

DocuSign Envelope ID: F44BE397-221A-4F0B-840B-52A8EAA64913 DocuSign Envelope ID: CD06F8BF-7BA1-4865-8AD6-CDF085D4307E

Protocol FH-58 (AD5NCOV-HIV2b) Version 1.5 September 10^th^, 2021

***6. STUDY PRODUCT AND ADMINISTRATION 55* 6.1 Description of Study Product 55 6.2. Product Oversight 57**

***7. SAFETY 59* 7.1 Definitions of an adverse event 59 7.2 Detection of adverse events 62 7.3 Participant contact of adverse events and serious adverse events 68 7.4 Treatment of adverse events 69**

***8. PARTICIPANT COMPLETION AND WITHDRAWAL 69* 8.1 Participant completion 69 8.2 Participant withdrawal 69**

***9. OUTCOME MEASURES AND STATISTICAL METHODS 71* 9.2 Study Population and Analysis Sets 71 9.3 Statistical Analysis 71**

**9.3.1 Demographics and Baseline Characteristics** 71 **9.3.2 Efficacy Analysis** 71 **9.3.4 Immunogenicity Analysis** 73 **9.3.5 Analysis of exploratory endpoints** 73

***10. DATA MANAGEMENT 74* 10.1 General Requirements 74 10.2 Electronic Data Capture System 75**

***11. STUDY SUSPENSION CRITERIA AND SAFETY MONITORING 75* 11.1 Study Suspension Criteria 75 11.2 Study Early Termination Criteria 75 11.3 Process of Suspension of Injection and/ or Study Modification 75**

***12. STUDY GOVERNANCE 76* 12.1 Study Steering Committee 76 12.2 Endpoint Review Committee 77**

Page **25** of **84**

DocuSign Envelope ID: F44BE397-221A-4F0B-840B-52A8EAA64913 DocuSign Envelope ID: CD06F8BF-7BA1-4865-8AD6-CDF085D4307E

Protocol FH-58 (AD5NCOV-HIV2b) Version 1.5 September 10^th^, 2021

**12.3 Independent Data Monitoring Committee 77 *13. ETHICAL AND REGULATORY CONSIDERATIONS 78* 13.1 Benefits/Potential Risks 78 13.2 Informed Consent Process and Documentation 78 13.3 Modification of the Protocol 78 13.4 Interruption of the Trial 79 13.5 Confidentiality of Data and Access to Participant Records 79 13.6 Monitoring 79 13.7 Auditing 79 13.8 Archiving 79 13.9 Stipends for Participation 79 13.10 Adverse Event Compensation and Insurance 79 13.11 Publication Policy 80 *14. ADMINISTRATIVE MATTERS 80* 14.1 Data Entry Instructions 80 14.2 Monitoring 80 14.3 Record Retention 81 14.4 Quality assurance 81 14.5 Posting of information on publicly available clinical trial registers 81 14.6 Provision of study results to investigators 82 *References 83***

**LIST OF FIGURES AND TABLES**

Figure 1. Procedure algorithm for efficacy contacts

Table 1. In-Person Visit

Table 2. Procedures for the group of participants assigned to placebo arm

Table 3. Adverse Event Reporting

Page **26** of **84**

DocuSign Envelope ID: F44BE397-221A-4F0B-840B-52A8EAA64913 DocuSign Envelope ID: CD06F8BF-7BA1-4865-8AD6-CDF085D4307E

Protocol FH-58 (AD5NCOV-HIV2b) Version 1.5 September 10^th^, 2021 Table 4. Time frames for submitting SAE to the Research Ethics Board………………………..

Page **27** of **84**

DocuSign Envelope ID: F44BE397-221A-4F0B-840B-52A8EAA64913 DocuSign Envelope ID: CD06F8BF-7BA1-4865-8AD6-CDF085D4307E

Protocol FH-58 (AD5NCOV-HIV2b) Version 1.5 September 10^th^, 2021

LIST OF ABBREVIATIONS

**AE** Adverse Event

**ATP** According-to-protocol

**BGTD**

**BMI**

**CCfV**

**CDISC**

**CI**

**COVID-19**

**CRO**

**DMP**

**ECMO**

**eCRF**

**EDC**

**ELISA**

Biologics and Genetic Therapies Directorate, Health Canada Body Mass Index

Canadian Center for Vaccinology

Clinical Data Interchange Standards Consortium Confidence Interval

Corona Virus Disease 2019

Contract Research Organization

Data Management Plan

Extra-corpeal membrane oxygen

Electronic Case Report Form

Electronic Data Capture System

Enzyme-linked-immunosorbent assays

**FDA** Food and Drug Administration, United States **FTiH** First time in human

**GCP**

**GMI**

**GMT**

**HAART**

**HIV**

**ICF**

Good Clinical Practice

Geometric Mean Increase

Geometric Mean Titer

Highly active anti-retroviral therapy

Human Immunodeficiency Virus Informed Consent Form

Page **28** of **84**

DocuSign Envelope ID: F44BE397-221A-4F0B-840B-52A8EAA64913 DocuSign Envelope ID: CD06F8BF-7BA1-4865-8AD6-CDF085D4307E

Protocol FH-58 (AD5NCOV-HIV2b) Version 1.5 September 10^th^, 2021

**ICH**

**ICS**

**IDMC IRB**

**IWRS MAE**

International Conference on Harmonization Intracellular Cytokine Staining

Independent Data Monitoring Committee Institutional Review Board

Interactive Web Response System Medically Attended AEs

**NRA** National Regulatory Authority

**PBMCs PI**

Peripheral Blood Mononuclear Cells Principal Investigator

**IRB** Research Ethics Board

**SAE**

**SAP**

**SARS-CoV-2 SC**

**SOP**

**SUSAR**

**TVC**

**VE**

**VP**

**WBC**

**WHO**

Serious Adverse Event

Statistical Analysis Plan

Severe Acute Respiratory Syndrome Coronavirus 2 Steering Committee

Standard Operating Procedure

Suspected Unexpected Serious Adverse Reaction

Total Vaccinated Cohort

Vaccine Efficacy

Viral particles

White Blood Cells

World Health Organization

Page **29** of **84**

DocuSign Envelope ID: F44BE397-221A-4F0B-840B-52A8EAA64913 DocuSign Envelope ID: CD06F8BF-7BA1-4865-8AD6-CDF085D4307E

Protocol FH-58 (AD5NCOV-HIV2b) Version 1.5 September 10^th^, 2021

GLOSSARY OF TERMS

**Adequate contraception:** Adequate contraception is defined as a contraceptive method with a failure rate of less than 1% per year when

used consistently and correctly and when applicable, in

accordance with the product label, such as:

● Abstinence from penile-vaginal intercourse, when this

is their preferred lifestyle,

● Oral contraceptives, either combined or progesterone

alone,

● Injectable progestogen,

● Implants of etonogestrel or levonorgestrel,

● Estrogen vaginal ring,

● Percutaneous contraceptive patches,

● Intrauterine device or intrauterine system,

● Male partner sterilization prior to the female subject’s

entry into the study, and this male is the sole partner

for that subject (information based on interview with

the participant on her medical history),

● Male condom combined with a vaginal spermicide

(foam, gel, film, cream or suppository),

● Male condom combined with a female diaphragm,

whether with or without a vaginal spermicide (foam,

gel, fil, cream, or suppository),

Adequate contraception does not apply to participants of

childbearing potential with same sex partners, when this

is their preferred and usual lifestyle.

**Adverse event:** Any untoward medical occurrence in a patient or clinical investigation participant, temporally associated with the

use of a medicinal product, whether or not considered

related to the medicinal product.

An adverse event (AE) can therefore be any unfavorable

and unintended sign (including an abnormal laboratory

finding), symptom, or disease (new or exacerbated)

temporally associated with the use of a medicinal product.

For marketed medicinal products, this also includes

failure to produce expected benefits (*i.e.* lack of efficacy),

Page **30** of **84**

DocuSign Envelope ID: F44BE397-221A-4F0B-840B-52A8EAA64913 DocuSign Envelope ID: CD06F8BF-7BA1-4865-8AD6-CDF085D4307E

Protocol FH-58 (AD5NCOV-HIV2b) Version 1.5 September 10^th^, 2021 abuse or misuse.

**Eligible:** Qualified for enrolment into the study based upon strict adherence to inclusion/exclusion criteria.

**Evaluable:** Meeting all eligibility criteria, complying with the

procedures defined in the protocol, and, therefore,

included in the according-to-protocol (ATP) analysis.

**Investigational product:**

**(Synonym of**

**Investigational**

**Medicinal Product)**

A pharmaceutical form of an active ingredient being tested or used as a reference in a clinical trial, including a product with a marketing authorization when used in a way different from the approved form, or when used for an unapproved indication, or when used to gain further information about an approved use.

**^Investigator’s Brochure:^** Compilation of the clinical and nonclinical data on the investigational product(s) that are relevant to the study of

the product(s) in human participants.

**^Participant:^**Term used throughout the protocol to denote an individual who has been contacted in order to participate

or participates in the clinical study, as a recipient of the

investigational product.

**^Randomization:^** Process of random attribution of treatment to participants in order to reduce bias of selection.

**Solicited adverse event:** Adverse events to be recorded as endpoints in the clinical study. The presence/occurrence/intensity of these events

is actively solicited from the participant or an observer

Page **31** of **84**

DocuSign Envelope ID: F44BE397-221A-4F0B-840B-52A8EAA64913 DocuSign Envelope ID: CD06F8BF-7BA1-4865-8AD6-CDF085D4307E

Protocol FH-58 (AD5NCOV-HIV2b) Version 1.5 September 10^th^, 2021

during a specified post-administration participant contact

period.

**Treatment:** Term used throughout the clinical study to denote a set of investigational product(s) or marketed product(s) or

intended to be administered to a participant, identified by

a unique number, according to the study randomization or

treatment allocation.

Page **32** of **84**

DocuSign Envelope ID: F44BE397-221A-4F0B-840B-52A8EAA64913 DocuSign Envelope ID: CD06F8BF-7BA1-4865-8AD6-CDF085D4307E

Protocol FH-58 (AD5NCOV-HIV2b) Version 1.5 September 10^th^, 2021

1. INTRODUCTION

**1.1. Background**

Since emerging in December 2019, the COVID-19 outbreak was deemed a Public Health Emergency of International Concern (PHEIC) by the WHO on 30 January 2020. On March 11^th^, 2020, COVID-19 was declared a global pandemic. As of Oct.20, 2020, there were over 42 million cases worldwide and over 1 million deaths in 185 countries/regions [1]. The COVID-19 pandemic has brought heavy economic pressure, medical burden, and severe harm to people's lives and health.

Currently, about 250 candidate vaccines against SARS-CoV-2 are in development worldwide, including mRNA vaccines, replicating or non-replicating viral vectored vaccines, DNA vaccines, autologous dendritic cell-based vaccine, and inactive virus vaccines [2].

One recently published trial in the UK investigated the use of a chimpanzee adenovirus-vectored vaccine (ChAdOx1 nCoV-19) expressing the SARS-CoV-2 spike protein [3]. This phase I/II single-blind, randomised, multi-site-controlled trial administered healthy adults (ages 18-55) with a 5 × 10^10^ viral particles (vp) dose of ChAdOx1 nCoV-19 or a control vaccine of meningococcal conjugate vaccine, at a 1:1 ratio. This candidate vaccine was found to be tolerable and immunogenic in healthy adults. Results demonstrated an increase in antibodies against SARS-CoV-2 spike protein in the ChAdOx1 nCoV-19 group, with levels peaking at Day 28 after vaccination, lasting until Day 56 in participants who received a single dose, and continuing to increase in participants who received a booster dose. An increase in cellular immune response was also demonstrated in the ChAdOx1 nCoV-19 group. Participants who received two doses of ChAdOx1 nCoV-19 demonstrated the highest humoral and cellular immune responses. The results of their phase I/II trial support moving forward with a large-scale phase III trial. Currently, there are 11 vaccines in phase III trials including three that have reported interim efficacy results.

**1.2. Investigational vaccine**

The 2019 novel coronavirus (SARS-CoV-2) is a positive non-segment single-stranded RNA virus, belonging to the Coronaviridae family of Nidovirales. There are six known coronaviruses in humans, including 229E and NL63 in the alpha genus, OC43 and HKU1 in the beta genus, Middle East respiratory syndrome-associated coronavirus (MERS-CoV), and severe acute respiratory syndrome-associated coronavirus (SARS- CoV).

The coronavirus isolated from the lower respiratory tract of patients with unexplained pneumonia in Wuhan is a new type of genus β coronavirus. Following the outbreak of severe acute respiratory syndrome coronavirus (SARS-CoV) in 2002 and the outbreak of Middle East Respiratory Syndrome coronavirus (MERS-CoV) in 2012, SARS-CoV-2 is the third highly pathogenic coronavirus.

Page **33** of **84**

DocuSign Envelope ID: F44BE397-221A-4F0B-840B-52A8EAA64913 DocuSign Envelope ID: CD06F8BF-7BA1-4865-8AD6-CDF085D4307E

Protocol FH-58 (AD5NCOV-HIV2b) Version 1.5 September 10^th^, 2021

The vaccine candidate Ad5-nCoV, intended to prevent COVID-19 caused by SARS-CoV-2, is jointly developed by CanSino Biologics Inc. and the Beijing Institute of Biotechnology. Using the replication-deficient human adenovirus type 5 as a vector, Ad5-nCoV has been developed

through recombinant virus construction, amplification and purification, and expresses the specific S protein of SARS-CoV-2. It is suggested that both humoral and cellular immune responses play an important role in protective immunity according to the results of pre-clinical, phase I and phase II studies.

Pre-clinical studies showed good immunogenicity (antibodies by ELISA and neutralization, cell mediated immunity) in mice, guinea pigs, rats, and cynomolgus monkeys. Protection by the Ad5- nCoV vaccine has been demonstrated in transgenic mice, ferrets and rhesus monkeys. Toxicology studies have been completed and have not demonstrated any concerns (see Investigator Brochure [10] for full details of the pre-clinical studies).

Phase I trials found that the Ad5-vectored COVID-19 vaccine was tolerable in healthy adults [5]. The study was designed and carried out as a dose-escalation, single-centre, open-label, non randomised clinical trial. 108 healthy adults participated in the phase I portion of the study and were divided into low, medium, and high-dose Ad5-nCoV vaccine groups. Participants received a dose on Injection visit. A total of 87% of participants reported at least 1 AE during the first 7 days after receiving the vaccine. The most common local AE was pain at the injection site, while fatigue, headache and fever were the most frequently reported systemic AE. Most reported AE were classified as mild to moderate and were self-limiting. However, there were 5 incidences of a Grade 3 fever in the high dose group. There was also higher occurrence of severe fever, fatigue, muscle pain, or joint pain, (which might be associated with viremia caused by Ad5 vector infection) in participants who received the of 1.5×10¹¹ viral particles. For this reason, the low (5×10¹⁰ vp per mL) and medium dose (1×10¹¹ vp per mL) were selected to progress to phase II trials. No SAE were recorded within 28 days of receiving the vaccine.

All three doses of the Ad5-nCoV vaccine demonstrated good immunogenicity in participants [5]. Anti-RBD antibodies were present in all groups after Day 14 and had at least a 4-fold increase in 97%, 94% and 100% of participants in the low, medium, and high doses respectively. Both ELISA and neutralizing antibodies increased significantly at 14 days post vaccination and peaked at 28 days post vaccination, in all three groups. A rapid T-cell response was observed in all groups by Day 14 as well.

On July 20^th^, 2020 the Phase II results of the Ad5-nCoV clinical trial were published. 508 healthy adults ≥ 18 years of age were randomized into three groups to receive Ad5-nCoV vaccine at either 1×10¹¹ vp per mL or 5×10¹⁰ vp per mL, or a placebo vaccine in a 2:1:1 ratio, respectively [2]. The main objectives of this study were to assess the immunogenicity and safety of receiving the Ad5-nCoV vaccine, and to determine the appropriate dose to be administered for the efficacy study (currently, phase III).

Page **34** of **84**

DocuSign Envelope ID: F44BE397-221A-4F0B-840B-52A8EAA64913 DocuSign Envelope ID: CD06F8BF-7BA1-4865-8AD6-CDF085D4307E

Protocol FH-58 (AD5NCOV-HIV2b) Version 1.5 September 10^th^, 2021

The data produced from the phase II clinical trial further supported the safety and tolerability of receiving an Ad5-nCoV vaccine in healthy adults [2]. There were no SAE recorded in any of the study groups within 28 days of vaccination. Participants in both the 1×10¹¹vp and 5×10¹⁰ vp groups commonly reported mild to moderate adverse reactions, when solicited by study staff. Injection site pain was the most frequently reported solicited reaction; 57% of the 1×10¹¹ vp dose and 56% of the 5×10¹⁰vp group reported pain within 14 days of vaccination. A total of 9% of participants in the 1×10¹¹vp had a Grade 3 adverse reaction, which was significantly higher than those who received 5×10¹⁰ vp vaccine (p=0.0011) or placebo (p=0.0004). The most commonly reported Grade 3 adverse reaction was a fever; however, fevers were self-limiting. There was no difference in the incidence of unsolicited adverse reactions between the three groups.

Both doses of Ad5-nCoV vaccine administered in the phase II study induced a robust immune response to SARS-CoV-2, with overall comparable immunogenicity between the doses [2]. Beginning Day 14, RBD-specific ELISA antibody responses were detectable in both groups and peaked at 656.5 (575.2-749.2) in the 1×10¹¹vp group, and 571.0 (467.6- 697.3) in the 5×10¹⁰vp group, on Day 28 post vaccination. While the placebo group showed no increase in antibodies from baseline, 96% of the 1×10¹¹vp group and 97% of the 5×10¹⁰vp group demonstrated seroconversion of the RBD-specific ELISA antibodies at Day 28. There was seroconversion of neutralising antibodies in 59% and 47% of participants in the 1×10¹¹vp and 5×10¹⁰ vp dose groups, respectively. There was a significant positive T-cell response in both the 1×10¹¹vp and 5×10¹⁰vp dose groups by Day 28, although the difference was not significant between the doses. A positive specific T-cell response, measured by IFNγ-ELISpot, was found in 90% of the 1×10¹¹ vp group and 88% of participants in the 5×10¹⁰ vp group. At Day 28, 95% of participants in the 1×10¹¹ viral particles dose group and 91% of the recipients in the 5×10¹⁰ viral particles dose group showed either cellular or humoral immune responses.

It was found that the 5×10¹⁰ vp group produced a similar immune response to the 1×10¹¹vp group, but with a better safety profile [2]. From the data produced in the Phase I and II trials, it has been determined that the best dose for the Ad5-nCoV vaccine is 5×10¹⁰ vp.

1**.3. Rationale for the study**

Largely attributable to globalization, without a prophylactic vaccine against SARS-CoV-2, COVID-19 will persist as a pandemic threat [2]. The few published clinical trials support the need for further researching of the efficacy of receiving vaccines that will protect against SARS CoV-2 infections [3].

This study is a phase IIb clinical trial to collect sufficient safety data to support the use of this candidate vaccine in HIV infected individuals.

Two doses of Ad5-nCoV at 5×10^10^vp will be administered to adults of 18 years of age and older, living with HIV, on stable treatment.

Page **35** of **84**

DocuSign Envelope ID: F44BE397-221A-4F0B-840B-52A8EAA64913 DocuSign Envelope ID: CD06F8BF-7BA1-4865-8AD6-CDF085D4307E

Protocol FH-58 (AD5NCOV-HIV2b) Version 1.5 September 10^th^, 2021

The Adenovirus type 5 platform has been successfully used by CanSino Biologics in its licensed Ebola virus vaccine. Two phase I clinical trials in China and one phase II clinical trial in Sierra Leone of Recombinant Ebola Virus Disease Vaccine (Adenovirus Type 5 Vector, rAd5) were conducted. The safety data are summarized below.

Between December 2014 and January 2015, a randomized, double-blinded, placebo-controlled, phase I clinical trial of single dose rAd5 Ebola vaccine was conducted in Jiangsu Province, China, to evaluate the safety, tolerability and immunogenicity in healthy people aged between 18 to 60 years. The vaccine was well tolerated with an adverse event reported in over 60% of participants after their primary immunization, and 71% of participants after the booster dose 6 months later [6, 7]. Local adverse reactions were pain, induration, and redness, swelling and itching. Systemic adverse reactions were fever, headache, fatigue, vomiting, diarrhea, myalgia, joint pain, sore throat and cough [6, 7]. Between March and August of 2015, a randomized, single center, open-label phase I clinical trial of rAd5 Ebola vaccine was conducted in Zhejiang Province, China, to evaluate its safety and immunogenicity in healthy Africans in China [8]. The safety results showed that solicited local adverse reactions were pain, swelling, induration and redness, mucosal damage, itching and rash at the injection site [8]. From October 2015, a randomized, single center, double-blinded, placebo-controlled phase II clinical trial of rAd5 Ebola vaccine was conducted in Sierra Leone [9], to evaluate its safety and immunogenicity in healthy Africans. The safety results showed that solicited local adverse reactions were mainly pain [9]. For full study details, please see articles referenced. Most of the adverse reactions were mild and self-limited. The above results indicated that the strength and extent of adverse reactions are acceptable and the rAd5 Ebola vaccine in healthy adults is tolerable and safe.

Immunogenicity of the rAd5 Ebola vaccine was demonstrated in all of the reported studies [6-9]. Higher antibody titers were demonstrated in participants who had low or absent pre-existing antibodies against adenovirus type 5; however, a significant immune response also occurred in individuals with pre-existing antibodies. These differences diminished after a booster dose of the rAd5 Ebola vaccine at 6 months [6-9].

Phase I and Phase II studies of the Adenovirus type 5 platform expressing SARS-CoV-2 spike protein have now been completed and demonstrate good safety and immunogenicity. A phase III trial to test the efficacy of the Ad5-nCoV vaccine is underway. A phase IIb study in persons living with HIV is warranted.

In its original version, the protocol limited inclusion to "people living with HIV on stable treatment and virologically suppressed...".

Likewise, the design was double-blind versus placebo in a 2:1 ratio.

The advancement of the national vaccination reduced the possibilities of access to the study under such design, for which this protocol amendment was proposed.

Page **36** of **84**

DocuSign Envelope ID: F44BE397-221A-4F0B-840B-52A8EAA64913 DocuSign Envelope ID: CD06F8BF-7BA1-4865-8AD6-CDF085D4307E

Protocol FH-58 (AD5NCOV-HIV2b) Version 1.5 September 10^th^, 2021

Volunteers initially included in the study will receive two doses of the vaccine and will continue their follow-up as established in the protocol.

**2. STUDY OBJECTIVES**

**2.1 Primary Objectives**

The primary objectives of the study are related to the safety and immunogenicity of the Ad5- nCoV in persons living with HIV.

There are four primary safety objectives.

1. Evaluate the incidence of solicited adverse reactions within 7 days after vaccination. 2. Evaluate the incidence of unsolicited adverse events within 28 days after vaccination. 3. Evaluate the HIV viral load 24 and 52 weeks after vaccination.

4. Evaluate the incidence of serious adverse events (SAE) and medically attended adverse events (MAE) within 52 weeks after vaccination in all participants.

The primary immunogenicity objectives are:

∙ Evaluate the seroconversion rate of S-RBD IgG antibody on Day 28, Day 84 and Week 24 and Week 52 after vaccination, measured by ELISA.

∙ Evaluate the immunogenicity of one versus two doses of the vaccine.

**2.2 Secondary Objectives**

**2.2.2 Secondary Safety Objective:**

∙ Evaluate the incidence of a decrease in CD4+ cell count by ≥20% at 24 and 52 weeks after vaccination.

∙ Evaluate changes in the CD4/CD8 ratio at 24 and 52 weeks compared to the basal value.

**2.2.3 Secondary Immunogenicity Objectives:**

1. Evaluate the GMT of S-RBD IgG antibody on Day 28, Day 84 and Week 24 and Week 52 after vaccination, measured by ELISA.

2. Evaluate the GMI of S-RBD IgG antibody on Day 28, Day 84 and Week 24 and Week 52 after vaccination, measured by ELISA.

3. Evaluate the seroconversion rate of pseudo-virus neutralizing antibody on Day 28, Day 84 and Week 24 and Week 52 after vaccination.

4. Evaluate the GMT of pseudo-virus neutralizing antibody on Day 28, Day 84 and Week

Page **37** of **84**

DocuSign Envelope ID: F44BE397-221A-4F0B-840B-52A8EAA64913 DocuSign Envelope ID: CD06F8BF-7BA1-4865-8AD6-CDF085D4307E

Protocol FH-58 (AD5NCOV-HIV2b) Version 1.5 September 10^th^, 2021

24 and Week 52 after vaccination.

5. Evaluate the GMI of pseudo-virus neutralizing antibody on Day 28, Day 84 and Week 24 and Week 52 after vaccination.

6. Evaluate the positive rate and level of IFN-γ, TNF, IL-4, IL-5, IL-13 stimulated by peptide pool of S protein on Day 28, Day 84 and Weeks 24 and Week 52 after vaccination, measured by intracellular cytokine staining (ICS) (in a subset of approximately 50 participants).

**2.2.4 Exploratory Objectives**

1. To evaluate the efficacy of two doses of Ad5-nCoV in preventing virologically confirmed (PCR positive) COVID-19 disease occurring 14 days and 28 days to 52 weeks after vaccination, regardless of severity.

2. To evaluate the efficacy of two doses of Ad5-nCoV in preventing virologically (PCR) or serologically (four-fold increase in SARS-CoV-2 anti-N IgG from pre-immunization to post symptom, defined as Day 21-28 post illness blood test, or pre-symptom and post symptom blood test) confirmed COVID-19 disease occurring 14 and 28 days to 52 weeks after vaccination, regardless of severity.

3. To evaluate the efficacy of two doses of Ad5-nCoV in preventing severe COVID-19 disease caused by SARS-CoV-2 infection from 14 and 28 days to 24 and 52 weeks after vaccination.

*Severe disease is defined as: 1) Clinical signs at rest indicative of severe systemic illness (respiratory rate ≥ 30 per minute, heart rate ≥ 125 per minute, SpO2 ≤*

*93% on room air at sea level or PaO2/FiO2 < 300 mm Hg), 2) Respiratory*

*failure (defined as needing high-flow oxygen, non-invasive ventilation,*

*mechanical ventilation or ECMO), 3) Evidence of shock (SBP < 90 mm Hg, DBP < 60 mm Hg, or requiring vasopressors), 4) Significant acute renal, hepatic, or*

*neurologic dysfunction, 5) Admission to an ICU.*

4. Evaluate the efficacy of two doses of Ad5-nCoV in preventing asymptomatic disease of COVID-19 (confirmed by N IgG antibody on week 52 after vaccination).

5. Evaluate the severity of COVID-19 cases among vaccine recipients (based on WHO or FDA criteria) as compared to the control group, to measure antibody-mediated disease enhancement (ADE).

6. Evaluate for any evidence of SARS-CoV-2 virus shedding in COVID-19 cases that occurred 28 days to 52 weeks after vaccination (detection of viral nucleic acid every 2 days after being confirmed).

7. Perform genotyping of SARS-CoV-2 virus isolates of COVID-19 cases that occurred 28 days to 52 weeks after vaccination.

8. Evaluate incidence of suspected but unconfirmed cases of COVID-19 (either because of negative or no tests).

Page **38** of **84**

DocuSign Envelope ID: F44BE397-221A-4F0B-840B-52A8EAA64913 DocuSign Envelope ID: CD06F8BF-7BA1-4865-8AD6-CDF085D4307E

Protocol FH-58 (AD5NCOV-HIV2b) Version 1.5 September 10^th^, 2021

**3. STUDY DESIGN**

**3.1 Summary**

This Phase IIb study is a multi-centre clinical trial; approximately 500 participants 18 years of age and older, living with HIV will be enrolled.

All participants will receive a single dose of the study vaccine (Ad5-nCoV) on First Injection Visit, and a second dose of Ad5-nCoV at Day 56 (Second Injection Visit) and will be followed to monitor vaccine candidate safety, immunogenicity (antibody response), and efficacy for a duration of 52 weeks. A subset of participants will also have cellular immunity measured after receiving the 5×10^10^ vp Ad5-nCoV vaccine (≥ 4 x10^10^ vp).

**Candidate Vaccine**: Ad5-nCoV

**Vaccination schedule: A double-dose schedule is used for all participants. The First dose at the Injection Visit when entering the study and the**

**Second dose at Day-56 Visit post baseline vaccination (Second**

**Injection Visit).**

**Data collection:** This clinical trial will use a single, designated Electronic Data Capture System (EDC) where participant electronic case report

forms (eCRFs) will be recorded.

**Safety Precautions**: Safety precautions will be outlined in detail in this section, as well as Section 7.

Blinding

**3.2 Study Groups**

A total of 500 participants will be enrolled to receive the vaccine. All participants will be monitored for solicited adverse events for 7 days, unsolicited adverse events for 28 days, SAEs and MAEs for the one year duration of the study, and antibody measurements at baseline, Day 28, Day 84, week 24, and week 52. A subset of 50 participants will have cellular immunity measured at those same intervals.

This protocol amendment provides for unblinding of participants who were entered into the study in the double-blind schedule. For participants who entered the double-blind schedule, the visit process will be as follows:

a) Sites will receive an unblinding list detailing participants who received placebo or study vaccine. Participants who received placebo will be invited to continue in the study under the

Page **39** of **84**

DocuSign Envelope ID: F44BE397-221A-4F0B-840B-52A8EAA64913 DocuSign Envelope ID: CD06F8BF-7BA1-4865-8AD6-CDF085D4307E

Protocol FH-58 (AD5NCOV-HIV2b) Version 1.5 September 10^th^, 2021

open-label study scheme and should make visits from V1.B (first vaccine) onwards, after signing the informed consent, completing visits on day 28 (V2.B), day 56 (second dose) (V3.B), day 84 (V4.B), week 24 (V5.B) and week 52 (V6.B). In this group of participants already enrolled, no antibody dosage collection will be performed at the V1B visit; the V1 values at study entry will be taken. The letter B is added to the visits to differentiate this group of participants who must reorganize the schedule of visits from the administration of the first dose of CoVID vaccine after the unblinding. The procedures for this group are detailed in Table 2.

b) Participants who, after the unblinding, are found to have received study vaccine at the initial visit, will complete the visits established by protocol together with the administration of dose 2 of the study (day 56) onwards, after signing the informed consent. In this group of participants there will be no changes in the nomenclature of the visits.

For both arms (placebo and study vaccine), efficacy contacts are restarted with the second injection visit.

**3.3 Sampling/Study Schedule**

All participants will have at least six planned in-person visits on First Injection Visit and Day 28, Day 56 (Second Injection Visit), Day 84, Week 24, and Week 52. The first visit, the Screening visit, will entail reviewing the ICF, completing a medical history of the participant, reviewing inclusion and exclusion criteria, collecting samples, performing HIV viral load and CD4/CD8 counts. At that vist (First Injection Visit), a urine pregnancy test in women of child-bearing potential will be performed (local site or laboratory) . The pre-vaccination blood drawn will be performed at First Injection Visit to obtain a viral load and a baseline CD4 count (CMH laboratory), a baseline SARS-CoV-19 antibody (Nexelis Laboratories), and baseline immunogenicity levels. Participants are required to remain at the study site for a minimum of 15 minutes after receiving the vaccine to monitor for any vaccine-related AE.

Participants will be given access to an e-diary or paper diary card to record solicited AE from First Injection Visit- day 7 and unsolicited AE from First Injection Visit-28. Participants will review their e-diary or diary card with study staff at their second in-person visit on Day 28. A new e-diary or paper diary card to record solicited and unsolicited AEs from Second Injection will be provided at Second Injection Visit- Day 56. The data in the completed e-diary or diary card will be reviewed and data will be collected at visit on Day 84.

Data collected from e-diaries and diary cards will be uploaded into the participant’s eCRF. Further details are outlined in Section 3.7 and 5.1.

All participants will be contacted on a weekly basis by the site (or through an automated message (email) with a link to answer the questions on whether or not they had SAE, MAE or COVID-19 symptoms) to be reminded to report any signs or symptoms of illness to study staff. The

Page **40** of **84**

DocuSign Envelope ID: F44BE397-221A-4F0B-840B-52A8EAA64913 DocuSign Envelope ID: CD06F8BF-7BA1-4865-8AD6-CDF085D4307E

Protocol FH-58 (AD5NCOV-HIV2b) Version 1.5 September 10^th^, 2021

reporting and monitoring of any participant illness are consistent with meeting the primary efficacy objective. If a participant becomes ill, they will be asked to immediately proceed to the site designated by their local study team where they will be assessed and treated as deemed appropriate. Participants will also receive a telephone call every 4 weeks to evaluate for the incidence of any SAE and MAE.

In-person visits on Screening visit and First Injection Visit, Day 28, Day 84, Week 24 and Week 52 will also be used for blood sample collection to assess for detailed immunogenicity response. Further details are outlined in Section 3.8 and 3.9.

A subset of approximately 50 participants will have additional immunologic testing performed, following the same visit schedule, to evaluate T-cell response to the Ad5-nCoV vaccine.

*If a participant demonstrates signs or symptoms of illness, an unplanned in-person visit may occur for evaluation and possible treatment. If, for any reason following a participant contact, the investigator would like to conduct an in-person visit with a participant, they may do so at their discretion*.

**3.4 Procedures**

Table 1. In-Person Visits

| **Intervention** |  | **Visit 1** | **Visit 2** | **Visit 3** | **Visit 4** | **Visit 5** | **Visit 6** |
| --- | --- | --- | --- | --- | --- | --- | --- |
|  |  | **Day 0**  **(Screeni**  **ng and**  **First**  **Injection Visit)** | **Day 28** | **Day 56 (Secon**  **d**  **Injecti**  **on**  **Visit)** | **Day 84** | **Week**  **24** | **Week**  **52** |
| Visit window |  |  | **+/-7**  **days** | **+/- 7**  **days** | **+/- 7**  **days** | **+/-14**  **days** | **+/-14**  **days** |
| Informed Consent |  | ● |  |  |  |  |  |
| Review Consent |  | ● | ● | **●** | **●** | ● | ● |
| Collect Demographic and  Participant Contact Information |  | ● |  |  |  |  |  |
| Blood sample collected for HIV viral load and CD4/CD8 counts |  | ● |  |  |  | ● | ● |
| Urine Pregnancy test- women of childbearing potential |  | ● |  | **●** |  |  |  |
| Review Inclusion and Exclusion criteria |  | ● |  | **●** |  |  |  |

Page **41** of **84**

DocuSign Envelope ID: F44BE397-221A-4F0B-840B-52A8EAA64913 DocuSign Envelope ID: CD06F8BF-7BA1-4865-8AD6-CDF085D4307E

Protocol FH-58 (AD5NCOV-HIV2b) Version 1.5 September 10^th^, 2021

| Medical History |  | ● |  | **●** |  |  |  |
| --- | --- | --- | --- | --- | --- | --- | --- |
| Check and record prior  concomitant medication and  vaccines |  | ● |  | **●** |  |  |  |
| Check and record intercurrent medical conditions |  | ● |  | **●** |  |  |  |
| Check Contraindications to vaccine |  | ● |  | **●** |  |  |  |
| Pre-injection vital signs |  | ● |  | **●** |  |  |  |
| Study group and treatment number allocation |  | ● |  |  |  |  |  |
| Screening conclusion |  | ● |  |  |  |  |  |
| Administration of Study Product |  | ● |  | **●** |  |  |  |
| Safety participant contact (Phone call or in-person visit) |  |  |  | **Every 4 weeks (Day 28, 56 and 84 is in person)** | | | |
| Efficacy participant contact  (message) *^(a)^* |  |  |  | **Weekly from Day 7- week 52**  **(visit window: +3 days)** | | | |
| Safety participant contact: in person visit |  | ● | ● | ● | ● | ● | ● |
| Paper diary provided to participant |  | ● |  | ● |  |  |  |
| E-diary or diary card reviewed with participant and data collected and uploaded to eCRF |  |  | ● |  |  |  |  |
| Immunogenicity participant  contact: in-person visit |  | ● | ● | ● |  | ● | ● |
| Blood Sample Collected for ELISA and neutralizing Antibody testing (~25mL) |  | ● | ● |  | ● | ● | ● |
| Blood Sample Collection for ICS testing (~30mL)^(b)^ |  | ● | ● |  | ● | ● | ● |
| Study conclusion |  |  |  |  |  |  | ● |

● *is used to indicate a study procedure.*

*^(a):^If it is preferable, a phone-call or text instead of email for an efficient participant contact is acceptable*

*^(b):^ In the subset of approximately 50 participants who will have cellular immunity measured* ***Detailed description of study procedures and interventions is outlined in Section 5.1***

Page **42** of **84**

DocuSign Envelope ID: F44BE397-221A-4F0B-840B-52A8EAA64913 DocuSign Envelope ID: CD06F8BF-7BA1-4865-8AD6-CDF085D4307E

Protocol FH-58 (AD5NCOV-HIV2b) Version 1.5 September 10^th^, 2021

**Table 2. Procedures for the group of participants assigned to placebo arm. In-person visit**

| **Intervention** |  | **Visit 1.B** | **Visit**  **2.B** | **Visit**  **3.B** | **Visit**  **4.B** | **Visit**  **5.B** | **Visit**  **6.B** |
| --- | --- | --- | --- | --- | --- | --- | --- |
|  |  | **Day 0**  **(First**  **Injection)** | **Day**  **28** | **Day**  **56**  **(Seco**  **nd**  **Inject**  **ion)** | **Day**  **84** | **Week 24** | **Week**  **52** |
| **Visit window** |  |  | **+/-7**  **days** | **+/- 7**  **days** | **+/- 7**  **days** | **+/-14**  **days** | **+/-14**  **days** |
| **Informed Consent** |  |  |  |  |  |  |  |
| **Review Consent** |  |  | **●** | **●** | **●** | **●** | **●** |
| **Collect Demographic and Participant Contact**  **Information** |  |  |  |  |  |  |  |
| **Blood sample collected for HIV viral load and**  **CD4/CD8 counts** |  |  |  |  |  | **●** | **●** |
| **Urine Pregnancy test**  **women of childbearing potential** |  | **●** |  | **●** |  |  |  |
| **Review Inclusion and**  **Exclusion criteria [2]** |  |  |  | **●** |  |  |  |
| **Medical History** |  | **●** |  | **●** |  |  |  |
| **Check and record prior concomitant medication and vaccines** |  | **●** |  | **●** |  |  |  |
| **Check and record**  **intercurrent medical**  **conditions** |  | **●** |  | **●** |  |  |  |
| **Check Contraindications to vaccine** |  | **●** |  | **●** |  |  |  |

Page **43** of **84**

DocuSign Envelope ID: F44BE397-221A-4F0B-840B-52A8EAA64913 DocuSign Envelope ID: CD06F8BF-7BA1-4865-8AD6-CDF085D4307E

Protocol FH-58 (AD5NCOV-HIV2b) Version 1.5 September 10^th^, 2021

| **Pre-injection vital signs** |  | **●** |  | **●** |  |  |  |
| --- | --- | --- | --- | --- | --- | --- | --- |
| **Study group and treatment number allocation** |  |  |  |  |  |  |  |
| **Screening conclusion** |  |  |  |  |  |  |  |
| **Administration of Study Product** |  | **●** |  | **●** |  |  |  |
| **Safety participant contact (Phone call or in-person visit)** |  |  |  | **Every 4 weeks (Day 28, 56 and 84 is in person)** | | | |
| **Efficacy participant contact (message) *^(a)^*** |  |  |  | **Weekly from Day 7- week 52 (visit window: +3 days)** | | | |
| **Safety participant contact: in-person visit** |  | **●** | **●** | **●** | **●** | **●** | **●** |
| **Paper diary card or e-diary provided to participant** |  | **●** |  | **●** |  |  |  |
| **E-diary or diary card**  **reviewed with participant and data collected and uploaded to eCRF** |  |  | **●** |  | ***●*** |  |  |
| **Immunogenicity**  **participant contact: in person visit** |  |  | **●** | **●** |  | **●** | **●** |
| **Blood Sample Collected for ELISA and neutralizing Antibody testing (~25mL)** |  |  | **●** |  | **●** | **●** | **●** |
| **Blood Sample Collection for ICS testing (~30mL)^(b)^** |  |  | **●** |  | **●** | **●** | **●** |
| **Study conclusion** |  |  |  |  |  |  | **●** |

Page **44** of **84**

DocuSign Envelope ID: F44BE397-221A-4F0B-840B-52A8EAA64913 DocuSign Envelope ID: CD06F8BF-7BA1-4865-8AD6-CDF085D4307E

Protocol FH-58 (AD5NCOV-HIV2b) Version 1.5 September 10^th^, 2021

**3.5 Efficacy participant contacts**

Efficacy participant contacts will occur for all participants on a weekly basis. Email or text messages will remind participants to report any signs or symptoms of infection to the study staff. These participant contacts can also be in-person, by phone, via the Internet or any other suitable method. Each contact needs to be recorded in the study electronic case report form.

Any reported COVID-19 relevant symptoms (e.g. fever, cough, shortness of breath, etc. as per WHO case definitions - see below) will trigger laboratory testing for SARS-CoV-2 infection. The SARS-CoV-2 nucleic acid test is performed with a throat or nasal swab and repeated within 3 days later if the result is negative. PCR SARS-CoV-2 tests will be done at Laboratorios CMH/Argenomics. Every effort will be made to verify positive PCR tests in a national or regional reference laboratory. Participants with a positive nasal/throat PCR will have it repeated at the local laboratory every two days in the first week (up to a maximum of 3 PCRs) and then weekly until negative. A serum specimen will be collected at the time of illness presentation and be repeated between 3 and 4 weeks later for SARS-CoV-2 anti-N IgG antibodies to be performed at Nexelis Laboratories for all subjects presented with suspected COVID-19 symptoms, regardless of the result of PCR SARS-CoV-2 test.


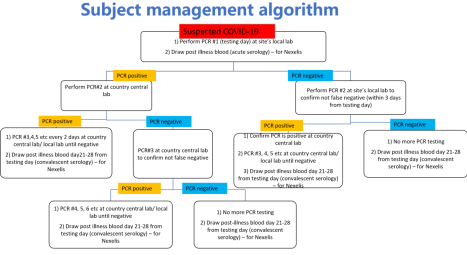
Figure 1. Procedure algorithm for efficacy contacts

After COVID-19 diagnosis, participants will be referred locally for management, as required. Data to determine whether these participants meet criteria for severe COVID-19 or if they receive antivirals that could modify the likelihood of severe COVID-19 will be collected. Study participant contact for COVID-19 disease is planned to last for at least 12 months.

Page **45** of **84**

DocuSign Envelope ID: F44BE397-221A-4F0B-840B-52A8EAA64913 DocuSign Envelope ID: CD06F8BF-7BA1-4865-8AD6-CDF085D4307E

Protocol FH-58 (AD5NCOV-HIV2b) Version 1.5 September 10^th^, 2021

Participants will have contacts via email, text, or phone message on Days 7, 14, 21, 35, 42, 49,, 63, 70, 77, 91, 98, 105, 112, 119, 126, 133, 140, 147, 154, 161,175, 182, 189, 196, 203, 210, 217, 224, 231, 238, 245, 252, 259, 266, 273, 280, 287, 294, 301, 308, 315, 322, 329, 336, 343, 350, and 357. Participant contacts on Days 56, 84, 112, 140, 196, 224, 252, 280, 308, and 336 will have a phone call, in addition to an email or text message. Day 28, 56, 84, 168 (week 24), and 364 (week 52) will be an in-person visit to accommodate the diary reviews and immunological testing.

*The visit window for Efficacy participant contact is +3 days.*

**Case definitions for surveillance:**

**Suspect case**

**Subject with any of the following symptoms (or signs) is reported as suspected case：**

**Fever, cough, dyspnoea & difficulty breathing, anosmia/ageusia, chills, myalgia, sore throat, prolonged fatigue, diarrhea, nausea, vomiting, headache, congested/runny nose, pneumonia, difficulty swallowing, loss of sense of smell and taste, and neurological events.**

**Probable case**

1. A suspect case for whom testing for the COVID-19 virus is inconclusive.

OR

2. A suspect case for whom testing could not be performed for any reason.

**Confirmed case**

A suspect case with laboratory confirmation of COVID-19 infection (including PCR positive result OR 4 folds or greater increase of anti N IgG at 4 folds or more raise between convalescence phase as compared to an acute phase), irrespective of clinical signs and symptoms.

**Endpoint case**

Confirmed cases are categorized into primary endpoint cases and secondary endpoint cases according to the time of onset after vaccination.

Primary endpoint: the participant with clinical symptom(s) that occurred not less than Confirmed cases with onset date ≥28 days after post- vaccination and the PCR test is positive.

Secondary endpoint: the participant with clinical symptom(s) that occurred not less than 14 days post-vaccination and the PCR test is positive; or 4 folds or greater increase of anti N IgG is detected after the occurrence of the clinical symptom(s).

Page **46** of **84**

DocuSign Envelope ID: F44BE397-221A-4F0B-840B-52A8EAA64913 DocuSign Envelope ID: CD06F8BF-7BA1-4865-8AD6-CDF085D4307E

Protocol FH-58 (AD5NCOV-HIV2b) Version 1.5 September 10^th^, 2021

Confirmed cases with onset date ≥28 days after vaccination.

Both primary and secondary endpoint cases need to be reported to the Endpoint Review Committee (ERC) for final review. A contact is a person who experienced any one of the following exposures during the 2 days before and the 14 days after the onset of symptoms of a probable or confirmed case:

1. Face-to-face contact with a probable or confirmed case within 1 meter and for more than 15 minutes;

2. Direct physical contact with a probable or confirmed case;

3. Direct care for a patient with probable or confirmed COVID-19 disease without using proper personal protective equipment;

OR

4. Other situations as indicated by local risk assessments.

*Note: for confirmed asymptomatic cases, the period of contact is measured as the 2 days before through the 14 days after the date on which the sample was taken which led to confirmation.*

**3.6 Immunogenicity In-Person Visits**

Participants will return to the study site on Day 28, Day 84, Week 24, and Week 52 for collection of venous blood samples for antibody testing. Approximately 25mL of serum will be collected for ELISA and neutralizing antibody testing on Day 28, Day 84, Week 24, and Week 52.

In the subset of 50 participants, approximately 30mL of whole blood will be collected on Day 28 and Day 84, and Week 24 and Week 52 for ELISpot testing and intracellular cytokine staining (ICS).

*The visit window for immunogenicity participant contact of Day28 and Day 84 is +/-7 days. The visit window for immunogenicity participant contact of Week 24 and Week 52 is +/-14 days.*

*Telephone visits and face-to-face visits at the same time point can be combined into one face-to face visit.*

**4. STUDY POPULATION**

The trial will recruit 500 participants, 18 years of age and older, living with HIV. Recruitment should support the generalizability of results, including by important characteristics such as age (including elderly). Participants must meet inclusion/exclusion criteria.

Page **47** of **84**

DocuSign Envelope ID: F44BE397-221A-4F0B-840B-52A8EAA64913 DocuSign Envelope ID: CD06F8BF-7BA1-4865-8AD6-CDF085D4307E

Protocol FH-58 (AD5NCOV-HIV2b) Version 1.5 September 10^th^, 2021

Participants will be enrolled at 4 study sites in Argentina. Depending on success of participant enrolment, as well as the COVID-19 attack rate, this list may be revised to include additional sites.

**4.1 Inclusion Criteria**

1. Adults of 18 years of age, and older, with confirmed HIV infection according to criterion 2.

2. A positive rapid test or ELISA and/or a plasma HIV-1 viral load >1000 copies/ml or rapid test or ELISA plus a positive Western Blot test. In patients on antiretroviral treatment, a medical history summary or prescription signed and stamped by the treating physician is acceptable. Able and willing (in the Investigator's opinion) to comply with all study requirements.

3. Willing to allow the investigators to discuss the volunteer's medical history with their General Practitioner/personal doctor and access all medical records when relevant to study procedures.

4. Healthy adults, or stable-healthy adults who may have a pre-existing medical condition that does not meet any exclusion criteria. A stable medical condition is defined as disease not requiring significant change in therapy or hospitalization for worsening disease during the 3 months before enrolment [4].

5. For subjects of childbearing potential only: Willingness to practice continuous effective contraception (see glossary) for 30 days prior to enrolment in the study, for 90 days after receiving vaccination during the study, and have a negative pregnancy test on the day(s) of screening/ vaccination (First Injection Visit and Second Injection Visit).

6. Males participating in this study who are involved in heterosexual sexual activity must agree to practice adequate contraception (see glossary) and refrain from donating sperm for 90 days after receiving the study vaccination.

7. Agreement to refrain from blood donation during the study.

8. Provide written informed consent.

**4.2 Exclusion Criteria**

1. Participation in any other COVID-19 prophylactic drug trials for the duration of the study. *Note: Participation in COVID-19 treatment trials is allowed in the event of hospitalization due to COVID-19. The study team should be informed as soon as possible*.

Page **48** of **84**

DocuSign Envelope ID: F44BE397-221A-4F0B-840B-52A8EAA64913 DocuSign Envelope ID: CD06F8BF-7BA1-4865-8AD6-CDF085D4307E

Protocol FH-58 (AD5NCOV-HIV2b) Version 1.5 September 10^th^, 2021

2. Planned receipt of any vaccine (licensed or investigational), other than the study intervention, within 14 days before and after study vaccination.

3. Prior receipt of an investigational or licensed vaccine likely to impact on the interpretation of the trial data (e.g. Adenovirus vectored vaccines, any coronavirus or SARS vaccines).

4. Administration of immunoglobulins and/or any blood products within the three months prior to the planned administration of the vaccine candidate.

5. Any confirmed or suspected immunosuppressive or immunodeficient state (other than living with HIV, on stable treatment, and virologically suppressed for at least 6 months); asplenia; recurrent severe infections and chronic use (more than 14 days) of immunosuppressant medication within the past 6 months. Topical steroids or short-term (course lasting ≤14 days) oral steroids are not an exclusion.

6. Active opportunistic infections or other AIDS-defining illness in the last six months.

7. History of allergic disease or reactions likely to be exacerbated by any component of Ad5- nCoV.

8. Any history of angioedema.

9. Any history of anaphylaxis to any vaccine component.

10. Pregnancy, lactation or willingness/intention to become pregnant within 90 days after receiving study vaccine.

11. Current diagnosis of or treatment for cancer (except basal cell carcinoma of the skin and cervical carcinoma in situ).

12. History of serious psychiatric condition likely to affect participation in the study.

13. Bleeding disorder (e.g. factor deficiency, coagulopathy or platelet disorder), or prior history of significant bleeding or bruising following IM injections or venepuncture.

14. Suspected or known current alcohol or drug dependency.

15. Severe and/or uncontrolled cardiovascular disease, respiratory disease, gastrointestinal disease, liver disease, renal disease, endocrine disorder and neurological illness (mild/moderate well-controlled comorbidities are allowed).

16. History of laboratory-confirmed COVID-19.

17. Continuous use of anticoagulants, such as coumarins and related anticoagulants (i.e. warfarin) or novel oral anticoagulants (i.e. apixaban, rivaroxaban, dabigatran and edoxaban).

18. Any other significant disease, disorder or finding which may significantly increase the risk to the volunteer because of participation in the study, affect the ability of the volunteer to

Page **49** of **84**

DocuSign Envelope ID: F44BE397-221A-4F0B-840B-52A8EAA64913 DocuSign Envelope ID: CD06F8BF-7BA1-4865-8AD6-CDF085D4307E

Protocol FH-58 (AD5NCOV-HIV2b) Version 1.5 September 10^th^, 2021

participate in the study or impair interpretation of the study data.

**4.3 Recruitment**

Participants will be recruited through invitations to persons in the study center’s participant database (who have previously indicated their willingness to be contacted regarding future studies) by mail and email, advertising using local print and radio media, social media, e-mail, notification through the study sites’ websites, through offices of local physicians, and in universities and health care settings. The numbers of persons who express interest in participating but are ineligible or declined participation, and reasons, will be recorded in the Screening Log.

**5. STUDY PROCEDURES AND INTERVENTIONS**

In-person visits will take place at selected study sites in Argentina, as well as through electronic (text message) and telephone calls. These facilities will be authorized by the institution to continue to do research related to COVID-19 disease during the pandemic. Scheduling during the pandemic will be arranged to minimize any social contact except with study staff. Study staff will screen potential participants for respiratory or other COVID-19 symptoms over the phone and again upon the participant’s arrival at the study center. Staff will wear medical masks at all times when interacting with participants to protect the participant and themselves from the potential of virus transmission in an asymptomatic individual, according to local institutional policies. These institution-wide precautions are meant to minimize the likelihood of a COVID-19 outbreak at the clinical trial site putting participants and staff at-risk, and to preserve the integrity of the study. These procedures will remain in place as long as the host institutional polices require them; study policies will continue to change to align with the host institution’s policies.

**5.1 Study Intervention Descriptions**

**All participants will be subject to the following procedure**s:

**● Informed consent**

The signed informed consent must be obtained before study participation. At each subsequent participant contact requiring an intervention (such as collection of a blood sample), the participant’s consent will be verified.

**● Check inclusion and exclusion criteria**

All inclusion and exclusion criteria will be checked at the screening in-person visit and will be reviewed at the second injection visit.

**● Collect demographic data and participant contact information**

Record demographic data such as date of birth, sex, gender, height, weight and race in the participant’s eCRF. Current email addresses and or phone numbers will be collected for each participant. It is important to have complete and accurate contact information for each participant as the majority of participant contacts will occur electronically. Contact information may also be used to remind participants of up-coming in-person visits.

Page **50** of **84**

DocuSign Envelope ID: F44BE397-221A-4F0B-840B-52A8EAA64913 DocuSign Envelope ID: CD06F8BF-7BA1-4865-8AD6-CDF085D4307E

Protocol FH-58 (AD5NCOV-HIV2b) Version 1.5 September 10^th^, 2021

**● Medical history**

Obtain the participant’s medical history by interview and/or review of the participant’s medical records and record any pre-existing conditions or signs and/or symptoms present in a participant prior to the first study injection in the eCRF. This will include reviewing any health condition that may prevent the participant from enrolling in the study, such as an unstable health condition. HIV treatment, clinical status, viral loads and CD4 counts will be reviewed.

**● HIV status, viral load, CD4 and CD8 counts**

At Screening visit, HIV status will be verified based on a previous serologic result or previous detectable viral load. Viral load samples, and CD4 and CD8 counts will be collected at Screening visit, and week 24 and week 52 visits. Screening visit value will be used as reference value for comparison with further values.

**● Baseline serology**

Approximately 25 mL of whole blood will be collected from each participant at First Injection Visit and separated for serum. The serum will be aliquoted. The first aliquot will be used to measure for baseline antibodies against SARS-CoV-2. Presence of pre-existing SARS-CoV-2 antibodies found in baseline serum will be used for a stratified

immunogenicity and efficacy sub-analysis. The second aliquot will be stored as a back-up. In participants who entered in the blind version and then were unblinded and vaccinated, baseline serology will not be performed again, taking the values of the baseline visit performed in the first visit.

**● Blood collection for ELISpot and ICS measurement**

In a subset of approximately 50 participants, approximately 30mL of whole blood will be collected from participants, in addition to the 25mL being collected for ELISA antibody analysis. TNF, IL-4, IL-5, IL-13 will be measured by ICS on first-injection day, Day 28, Day 84 and Weeks 24 and 52 after vaccination. PBMC will be shipped on dry ice to Nexelis Laboratories (Canada).

**● Check contraindications, warnings and precautions to injection**

Contraindications, warnings and precautions to injection must be checked at the beginning of each Injection Visit.

**● Urine Pregnancy Test/Birth Control**

Women of child-bearing age will be asked to perform a urine pregnancy test before each Injection Visit. The result of the urine pregnancy test must be negative in order to proceed with vaccine administration. In addition, participants who are able to become pregnant or could impregnate a partner are required to have used approved contraception least 30 days prior to the study vaccination and for 90 days after the study vaccination.

**● Assess pre-injection vital signs (body temperature, blood pressure, pulse rate and respiratory rate)**

Body temperature of all participants needs to be measured prior to any study product administration. Body temperature may be measured by any method (oral, axillary). If the

Page **51** of **84**

DocuSign Envelope ID: F44BE397-221A-4F0B-840B-52A8EAA64913 DocuSign Envelope ID: CD06F8BF-7BA1-4865-8AD6-CDF085D4307E

Protocol FH-58 (AD5NCOV-HIV2b) Version 1.5 September 10^th^, 2021

participant has fever (fever is defined as temperature ≥38.0° C orally or ≥37.8 axillary) on the day of injections, the injection visit will be rescheduled within 1 week.

**● Screening conclusion**

Participants will be deemed eligible to participate upon reviewing medical history and inclusion and exclusion criteria. This will occur prior to vaccination at First and Second Injection Visit.

**● Check and record prior and concomitant medication/vaccines**

Prior medications and concomitant medication/injection must be checked and recorded in the eCRF. Prior medications should include any medication taken by the participant within 14 days prior to screening. Participants will be asked to avoid over-the-counter medications such as antipyretics (e.g., acetaminophen) and anti-inflammatory medications (e.g., ibuprofen, naproxen) in the 12 hours before study vaccine receipt but will be allowed to take these over-the-counter medications as needed to treat fever or other adverse events after vaccination. Usage of these over-the-counter medications will be recorded as concomitant medications and linked to the adverse event collected as solicited or unsolicited events according to the symptom.

**● Check and record intercurrent medical conditions**

Any medical conditions that have arisen are recorded in the eCRF.

**● Treatment number allocation**

The treatment number will be allocated within each step. The number of each administered treatment must be recorded in the eCRF.

**● Injection of study vaccine**

After completing all prerequisite procedures prior to the active vaccine injection, one dose of the assigned vaccine will be administered IM, in the deltoid muscle, preferable of the non dominant arm. If the investigator or delegate determines that the participant’s health on the day of administration temporarily precludes administration, the visit will be rescheduled within 1 week. There will be a 15-minute wait after each vaccination to monitor for any rare anaphylactic reaction. A second injection, of active vaccine Ad5-nCoV, will be administered to all participants on Day 56.

**● Safety participant contacts**

Every 4 weeks there will be telephone calls (Weeks 8-52) to continuously collect any SAE and MAE data. Participants can report any AE they experience at any time during the study period but only SAE and MAE will be entered into the eCRF. Safety participant contacts will occur at First Injection Visit (for 15 minutes post-vaccination), Day 28 (to collect the diary), Day 56 (Second Injection Visit), Day 84 (to collect the diary), Month 4, 5, 6, 7, 8, 9, 10, 11, and 12. Safety participant contacts will be a telephone call for Months 2, 3, 4, 5, 7, 8, 9, 10 and 11, and as an in-person visit on Month 1 (Day 28), Month 2 (Day 56), Month 3 (Day 84), Month 6 (Week 24), and Month 12 (Week 52).

**● E-diary and Diary Cards**

Page **52** of **84**

DocuSign Envelope ID: F44BE397-221A-4F0B-840B-52A8EAA64913 DocuSign Envelope ID: CD06F8BF-7BA1-4865-8AD6-CDF085D4307E

Protocol FH-58 (AD5NCOV-HIV2b) Version 1.5 September 10^th^, 2021

Participants will be provided with access to an e-diary or a paper diary card and a thermometer, and instructions about how to use them. The use of an e-diary instead of a paper diary card is dependent on the study site. Participants will be asked to record solicited AE for 7 days after receiving the vaccination, and unsolicited AE for 28 days, in their diary. The completed e-diary and diary cards will be reviewed, and data collected at the 28-Day safety in-person visit. A new diary will be provided at Day 56 and collected at Day 84. Non completion of the e-diary or diary cards will be investigated with the participant through telephone call(s) or any other convenient procedure. All data collected from the e-diary and diary cards will be uploaded into the eCRF.

**● Efficacy participant contacts**

All participants will have efficacy participant contacts weekly for 52 weeks following vaccination. There will be approximately 52 electronic efficacy participant contacts. All efficacy participant contacts will be text messages, although other electronic method such as by phone, e-email, the Internet is suitable, if preferred. Each participant contact needs to be recorded in the study electronic case report form. The purpose of these participant contacts is to serve as a reminder to participants to report any symptoms of illness they may be experiencing, regardless of severity. Any possible COVID-19 relevant symptoms (e.g., fever, cough, shortness of breath, etc., according to WHO case definitions; see Section 3.8) will trigger laboratory testing for SARS-CoV-2 infection.

**● Illness participant contact visits (Confirmed or suspected infection during study)** Participants will be provided detailed instructions regarding the signs and symptoms of COVID-19 disease at each participant contact and will be instructed to seek medical attention and notify study staff should symptoms occur filling the illness visit form and completing the eCRF. The form should be updated and sent and sent when the case meets the definition of severe COVID, and when the case resolves. Symptoms of concern include cough, fever, diarrhea, prolonged fatigue, respiratory symptoms, pneumonia, difficulty swallowing, loss of sense of smell and neurological events. For symptoms of interest, please refer to 3.8 efficacy participant contact.

During the observation period of the study, if any of these symptoms develop in a participant, he/she should immediately follow local procedures for care of suspected COVID-19 illness and contact the study team. Specimen collection for the study will follow the study illness visit algorithm (see Figure 2). The participant’s nasopharyngeal/throat swab will be collected and tested for COVID-19 infection in either a local clinical or study site laboratory. If negative, the test will be repeated in 3 days. Repeat nasopharyngeal/throat swabs will be taken every 2 days until a negative sample is obtained (if possible). A serum taken at the time of illness presentation and 2-3 weeks later will be collected for SARS-CoV 2 anti-N IgG antibodies to be performed in the study central laboratory. A serum taken at the time of illness presentation and 21-28 days later will be collected for SARS-CoV-2 anti N IgG antibodies to be performed at Nexelis laboratories for all subjects presented with suspected COVID-19 symptom, regardless of the outcome of PCR SARS-CoV-2 test. If a

Page **53** of **84**

DocuSign Envelope ID: F44BE397-221A-4F0B-840B-52A8EAA64913 DocuSign Envelope ID: CD06F8BF-7BA1-4865-8AD6-CDF085D4307E

Protocol FH-58 (AD5NCOV-HIV2b) Version 1.5 September 10^th^, 2021

COVID-19 infection is found during the study, a case investigation will be undertaken according to locally recommended procedures. Participants who develop COVID-19 infection post-vaccination will be carefully monitored for vaccine related disease

enhancement in collaboration with their primary physician who will be encouraged to obtain specimens in accordance with the Brighton Collaboration definition (Munoz, 2020). This monitoring may include testing for C-reactive protein (CRP), ferritin, and procalcitonin, biomarkers that have been associated with predicting more severe disease outcome in infected patients. Repeat nasopharyngeal/throat swabs will be taken every 2 days (by 3 times) and weekly later until a negative sample is obtained. Participants who develop COVID-19 disease will continue to be followed for other study outcomes by telephone while in isolation, and at the study center once recovered and released from isolation by public health officials.

**● Immunogenicity in-person visit**

**o Blood collection for ELISA Antibody measurement**

**o** Participants will donateapproximately 25mL of whole blood on First Injection Visit, Day 28, Day 84 and Week 24, and Week 52, as described above. Whole

blood will be separated into serum, and then aliquoted. These in-person visits will occur at participants’ local study site. A baseline antibody titer against SARS

CoV-2 will be established from the blood sample collected on First Injection

Visit, as outlined in the “baseline serology” description. In addition, these samples will also be used to evaluate for additional immunogenicity objectives. Serum

collected will be analyzed for the seroconversion rate of S-RBD IgG antibody,

GMT of S-RBD IgG antibody, GMI of S-RBD IgG antibody, seroconversion rate of pseudo-virus neutralizing antibody, GMT of pseudo-virus neutralizing

antibody, and the GMI of pseudo-virus neutralizing antibody at Day 28, Day 84,

Week 24, and Week 52. The second aliquot will be shipped by the central

laboratory to Nexelis Laboratories, where it will be stored as a back-up.

**o Blood collection for ELISpot and ICS measurement**

In a subset of approximately 50 participants, approximately 30mL of whole blood will be collected from participants on First Injection Visit, Day 28, Day 84 and

Week 24, and Week 52, as described above. TNF, IL-4, IL-5, IL-13 will be

measured by ICS on Day 28, Day 84 and Weeks 24 and 52 after vaccination.

PBMC will be shipped on dry ice to Nexelis Laboratories (Canada).

**● Final serology**

Participants will have an in-person visit on Month 12. Approximately 25 mL of whole blood will be collected from each participant and separated for serum into two aliquots. The first aliquot will measure antibodies against SARS-CoV-2, at Nexelis laboratories. The second aliquot will be shipped by Nexelis laboratories to the Sponsor, where it will be stored as a back-up. The one-year antibody levels measured against SARS-CoV-2 will be compared to

Page **54** of **84**

DocuSign Envelope ID: F44BE397-221A-4F0B-840B-52A8EAA64913 DocuSign Envelope ID: CD06F8BF-7BA1-4865-8AD6-CDF085D4307E

Protocol FH-58 (AD5NCOV-HIV2b) Version 1.5 September 10^th^, 2021

the participant’s baseline levels to evaluate the exploratory efficacy analysis against asymptomatic infection.

*The visit window for immunogenicity in-person visits of Day 28 and Day 84 is /-7 days. The visit window for immunogenicity in-person visits of Week 24, and Week 52 is +/- 14 days*

**5.2 Study conclusion**

Upon study conclusion (52 weeks post-vaccination), the investigators will:

**●** Review data collected to ensure accuracy and completeness

**●** Complete the Study Conclusion screen in the eCRF.

**6. STUDY PRODUCT AND ADMINISTRATION**

**6.1 Description of Study Product**

Recombinant Novel Coronavirus Vaccine (Adenovirus Type 5 Vector). This vaccine, Ad5- nCoV, is a replication-defective recombinant human type 5 adenovirus expressing novel coronavirus S protein, which is produced by viral amplification in HEK293SF-3F6 cells, purification and formulation with addition of proper excipients. The product is used for prevention from infection caused by novel coronavirus.

**Active Ingredients:** Replication-defective recombinant human type 5 adenovirus expressing S protein of novel coronavirus

**Excipients:** Mannitol, sucrose, sodium chloride, magnesium chloride, polysorbate 80, HEPES, and glycerin

**Packaging**: The vaccine is contained in a vial.

**Specification:** 0.5 mL/vial

**Dosage** 5x10^10^ vp (≥4 x 10^10^ vp)

* Explanatory note: The vials contain a volume of 0.5 ml. The indication is to administer this amount. The manufacturer of the vaccine has advised that a volume between 0.4 ml and 0.5 ml per dose is permitted since it has been calculated that the 0.4 ml extractable volume of each vial in the lot involved in this study (Lot NCOV202103006) has met the requirements of the study based on good vaccination practices. The vaccines used in this study have passed the quality control procedure during the filling process and the fill volume of the vaccines meets the quality standards.

**Shelf Life**: Tentatively 24 months

**Storage:** This vaccine should be stored and transported at 2-8℃.

Page **55** of **84**

DocuSign Envelope ID: F44BE397-221A-4F0B-840B-52A8EAA64913 DocuSign Envelope ID: CD06F8BF-7BA1-4865-8AD6-CDF085D4307E

Protocol FH-58 (AD5NCOV-HIV2b) Version 1.5 September 10^th^, 2021

**Administration:** Intramuscular injection in the deltoid muscle of the upper arm.

**Schedule:** A double-dose of active vaccine is planned for all participants. A second dose of active vaccine is planned for all participants 56 days after the first

Injection.

**Manufacturer**: CanSino Biologics Inc.

185 South Ave., TEDA West District, Tianjin, China

Postal code: 300462

Tel: 86-022-58213600

Fax: 86-022-58213677

Website: www.cansinotech.com

**Developers:** CanSino Biologics Inc.

Beijing Institute of Biotechnology

**Contraindications to the Study Vaccine**

(1) Allergic reaction to any component of this vaccine.

(2) Acute illness, acute phase of chronic disease, and fever.

(3) Pregnant and lactating women.

(4) People with thrombocytopenia or hemorrhagic diseases, immune-suppressive therapy or immune dysfunction, and uncontrolled seizure disorder and other progressive neurological disorders.

**Warnings and Precautions**

**1.** This vaccine is strictly prohibited to be administered by intravascular injection. **2.** Epinephrine and other drugs as well as equipment should be in place when the vaccine is used, in case emergency treatment of severe allergic reactions will be

needed. Those who are immunized with this vaccine should be observed for at

least 30 minutes at the site.

**3.** As with all vaccines, this vaccine may not produce 100% protection in the

vaccinated population.

**4.** The vaccine must be stored in the place not accessible by children.

**5.** The vaccine should be shaken before injection. It should not be used under

following circumstances: presence of foreign objects, cracked vaccine syringe,

unclear or expired label, or any other abnormal appearance of the vaccine.

**6.** No disinfectant in contact with the vaccine when injection.

Page **56** of **84**

DocuSign Envelope ID: F44BE397-221A-4F0B-840B-52A8EAA64913 DocuSign Envelope ID: CD06F8BF-7BA1-4865-8AD6-CDF085D4307E

Protocol FH-58 (AD5NCOV-HIV2b) Version 1.5 September 10^th^, 2021

**6.2. Product Oversight**

**Product accountability**

A product accountability log will be maintained by study staff to document the number of vials of product received and the disposition of each dose.

**Replacement doses**

Replacements will be from the study stock.

**Return of Unused Products**

Following verification of product accountability, all empty vials will be destroyed as per site policy. At the end of the study, all unused clinical trial vaccine material will be returned to the sponsor or disposed of according to the site’s standard operating procedure.

**Dosage and administration of study products by the study nurse**

A study nurse at each clinical site will be responsible for the administration of the IM vaccine into the patient’s deltoid muscle. Products will be administration according to the prompts in the IWRS system.

**Contraindications to administration of the investigational product at First Injection Day**

The following events constitute contraindications to administration of the study products at that point in time. If any contraindication is present at the time scheduled for injection, the participant may be injected at a later date, within the time window specified in the protocol, or withdrawn at the discretion of the investigator.

● Acute disease and/or fever at the time of administration.

● Fever is defined as temperature ≥38.0°C, taken orally.

● Participants with a minor illness (such as mild diarrhea, mild upper respiratory infection) without fever can be administered the product.

**Contraindications to administration of the study product at Day 56 Second Injection Day**

● Acute disease and/or fever at the time of administration.

● Suspicion of COVID19 case due to symptoms.

● Fever (≥ 39.0 °C) lasting at least 72 hs after the first injection.

● Adverse events related to the study product (severe or serious).

**Concomitant medications/products and concomitant administration**

At each participant contact over the phone or in-person, the investigator should question the participant about any medication/product taken and injection received by the participant.

**Recording of prior and concomitant medications/products and concomitant administration**

Page **57** of **84**

DocuSign Envelope ID: F44BE397-221A-4F0B-840B-52A8EAA64913 DocuSign Envelope ID: CD06F8BF-7BA1-4865-8AD6-CDF085D4307E

Protocol FH-58 (AD5NCOV-HIV2b) Version 1.5 September 10^th^, 2021

The following prior and concomitant medications/products/vaccines must be recorded in the eCRF if administered during the indicated recording period:

● All concomitant medications/products, except vitamins and dietary supplements, administered from 14 days prior to screening up to 28 days following any dose of study product.

● Any concomitant vaccination administered in the period starting from screening up to study end.

● Prophylactic medication (*i.e.* medication administered in the absence of ANY symptom and in anticipation of a reaction to the injection). *E.g.* an anti-pyretic is considered to be prophylactic when it is given in the absence of fever and any other symptom, to prevent fever from occurring.

● Any concomitant medication/product/vaccine relevant to a SAE^*^or administered at any time during the study period for the treatment of a SAE^*^, or administered at any time during the study for treatment of an adverse event leading to study termination, or treatment of an adverse event requiring a medically attended visit.

*^*^SAEs that are required to be reported per protocol.*

**Concomitant medications/products/vaccines that may lead to the elimination of a participant from according-to-protocol (ATP) analyses**

The use of the following concomitant medications/products/vaccines will not require withdrawal of the participant from the study but may determine a participant’s evaluability in the according to-protocol (ATP) analysis:

● Any investigational or non-registered product (drug or vaccine) other than the study product(s) used during the study period.

● A vaccine not foreseen by the study protocol administered during the period starting from 14 days before injection and ending 14 days after a study vaccine injection. It should be noted that in case an emergency mass vaccination for a unforeseen public health threat other than COVID-19 is organized by the public health authorities, outside the routine immunization program, the time period described above can be reduced if necessary for that vaccine provided it is authorized by the regulatory authority and used according to its package insert and according to the local governmental recommendations and provided a written approval of the Sponsor is obtained.

● Immunosuppressants or other immune-modifying drugs administered chronically (*i.e* more than 14 days) during the study period (for corticosteroids, this will mean prednisone ≥ 20 mg/day, or equivalent). Inhaled and topical steroids are allowed.

● Long-acting immune-modifying drug administered at any time during the study period (*e.g.* infliximab).

Page **58** of **84**

DocuSign Envelope ID: F44BE397-221A-4F0B-840B-52A8EAA64913 DocuSign Envelope ID: CD06F8BF-7BA1-4865-8AD6-CDF085D4307E

Protocol FH-58 (AD5NCOV-HIV2b) Version 1.5 September 10^th^, 2021

● Immunoglobulins and/or any blood products administered during the study period.

**Intercurrent medical conditions that may lead to elimination of a participant from ATP analyses**

At each participant contact subsequent to the First Injection Visit, it must be verified if the participant has experienced or is experiencing any intercurrent medical condition. If it is the case, the condition(s) must be recorded in the eCRF.

Participants may be eliminated from the ATP cohort for immunogenicity if, during the study, they incur a condition that has the capability of altering their immune response (*e.g.* confirmed influenza infection) or if they become diagnosed with an immunological disorder.

**7. SAFETY**

A primary objective of this study focuses on the tolerability and safety of receiving two doses of 5×10^10^vp Ad5-nCoV vaccine. This will be measured by the incidence of SAE and MAE within 52 weeks of receiving the vaccine and solicited AE (*local* injection site reactions and *general* reactions within the seven days following vaccination) and unsolicited AE within 28 days after vaccination.

The investigator or site staff is/are responsible for the detection, documentation and reporting of events meeting the criteria and definition of an AE, MAE, or SAE as provided in this protocol. Each participant will be instructed to contact the investigator immediately should they manifest any signs or symptoms they perceive as serious.

Table 3. Adverse Event Reporting:

| **Participants** | **30 min**  **observation** | **First Injection**  **Visit- Day 7** | **Day 8-28** | **Beyond day 29 until week 52** |
| --- | --- | --- | --- | --- |
| All | Post injection AE or SAE  only | Solicited AEs and unsolicited AEs  SAE and MAE | Any unsolicited AE only  SAE and MAE | SAE and MAE only |

**7.1 Definitions of an adverse event**

An **AE** is “an untoward medical occurrence in a clinical investigation subject, temporally associated with the use of a medicinal product, whether or not considered related to the medicinal product.” A medical attended AE (MAE) is an AE that requires a visit with the healthcare system. A serious AE (SAE) is an untoward medical occurrence that results in death, is life-threatening (that is, there was a risk of death at the time of the event), requires

Page **59** of **84**

DocuSign Envelope ID: F44BE397-221A-4F0B-840B-52A8EAA64913 DocuSign Envelope ID: CD06F8BF-7BA1-4865-8AD6-CDF085D4307E

Protocol FH-58 (AD5NCOV-HIV2b) Version 1.5 September 10^th^, 2021

hospitalization or prolongation of existing hospitalization occurs, results in disability/incapacity, or is a congenital anomaly/birth defect in the offspring of a study subject.

An AE can therefore be any unfavourable and unintended sign (including an abnormal laboratory finding), symptom, or disease (new or exacerbated) temporally associated with the use of a medicinal product.

Examples of an AE include:

● Significant or unexpected worsening or exacerbation of the condition/indication under study.

● Exacerbation of a chronic or intermittent pre-existing condition including either an increase in frequency and/or intensity of the condition.

● New conditions detected or diagnosed after investigational product administration even though they may have been present prior to the start of the study.

● Signs, symptoms, or the clinical sequelae of a suspected interaction.

● Signs, symptoms, or the clinical sequelae of a suspected overdose of either investigational product or a concurrent medication (overdose *per se* should not be reported as an

AE/SAE).

● Signs, symptoms temporally associated with product administration.

● Significant failure of expected pharmacological or biological action.

● Pre- or post-treatment events that occur as a result of protocol-mandated procedures (i.e. invasive procedures, modification of participant’s previous therapeutic regimen).

AEs to be recorded as endpoints (solicited AEs) are described below.

All other AEs will be recorded as unsolicited AEs.

Examples of an AE DO NOT include:

● Medical or surgical procedures (e.g. endoscopy, appendectomy); the condition that leads to the procedure is an AE/SAE.

● Situations where an untoward medical occurrence did not occur (e.g. social and/or convenience admission to a hospital, admission for routine examination).

● Anticipated day-to-day fluctuations of pre-existing disease(s) or condition(s) present or detected at the start of the study that do not worsen.

● Pre-existing conditions or signs and/or symptoms present in a participant prior to the first study injection. These events will be recorded in the medical history.

**Solicited adverse events**

A ***solicited* adverse event** (AE) is a pre-specified outcome that the participant is asked to record as present or not, and if present, to apply an intensity rating. Solicited AE are collected daily from Days 0 to 7 following administration of the study vaccine. An *unsolicited* event is one that

Page **60** of **84**

DocuSign Envelope ID: F44BE397-221A-4F0B-840B-52A8EAA64913 DocuSign Envelope ID: CD06F8BF-7BA1-4865-8AD6-CDF085D4307E

Protocol FH-58 (AD5NCOV-HIV2b) Version 1.5 September 10^th^, 2021

the participant identifies when asked in a non-leading manner if there have been any changes in their health since the last participant contact.

Solicited AEs (Solicited local (injection-site) and general AEs) occurring during the 7-day participant contact period after injection will be recorded.

**Solicited local (injection-site) adverse events**

The following local (injection-site) AEs will be solicited:

**●** Pain at injection site

**●** Redness at injection site

**●** Swelling at injection site

**Solicited general adverse events**

The following general AEs will be solicited:

**●** Drowsiness

**●** Fever

**●** Headache

**●** Nausea

**●** Diarrhea

**●** Vomiting

**●** Generalized muscle aches

*Note: Temperature will be recorded in the evening. Should additional temperature measurements be performed at other times of day, the highest temperature will be recorded in the eCRF.*

**Definition of a serious adverse event**

An SAE is any untoward medical occurrence that:

a. Results in death,

b. Is life-threatening,

Note: The term ‘life-threatening’ in the definition of ‘serious’ refers to an event in which the participant was at risk of death at the time of the event. It does not refer to an event, which hypothetically might have caused death, had it been more severe.

c. Requires hospitalisation or prolongation of existing hospitalisation,

Note: In general, hospitalisation signifies that the participant has been admitted at the hospital or emergency ward for observation and/or treatment that would not have been appropriate in the physician’s office or in an out-patient setting. Complications that occur during

Page **61** of **84**

DocuSign Envelope ID: F44BE397-221A-4F0B-840B-52A8EAA64913 DocuSign Envelope ID: CD06F8BF-7BA1-4865-8AD6-CDF085D4307E

Protocol FH-58 (AD5NCOV-HIV2b) Version 1.5 September 10^th^, 2021

hospitalisation are also considered AEs. If a complication prolongs hospitalisation or fulfils any other serious criteria, the event will also be considered serious. When in doubt as to whether ‘hospitalisation’ occurred or was necessary, the AE should be considered serious.

Hospitalisation for elective treatment of a pre-existing condition (known or diagnosed prior to informed consent signature) that did not worsen from baseline is NOT considered an AE.

d. Results in disability/incapacity,

Note: The term disability means a substantial disruption of a person’s ability to conduct normal life functions. This definition is not intended to include experiences of relatively minor medical significance such as uncomplicated headache, nausea, vomiting, diarrhoea, influenza like illness, and accidental trauma (e.g. sprained ankle) which may interfere or prevent everyday life functions but do not constitute a substantial disruption.

e. Congenital anomaly/birth defects,

Note: Medical judgment should be exercised in deciding whether reporting is appropriate in other situations, such as important medical events that may not be immediately life threatening or result in death or hospitalisation but may jeopardise the participant or may require medical or surgical intervention to prevent one of the other outcomes listed in the above definition. These should also be considered serious. Examples of such events are invasive or malignant cancers, intensive treatment in an emergency room or at home for allergic bronchospasm, congenital anomaly/birth defects, blood dyscrasias, or convulsions that do not result in hospitalisation.

**Clinical laboratory parameters and other abnormal assessments qualifying as adverse events or serious adverse events**

Although routine clinical laboratory monitoring is not a part of this phase IIb trial, a participant may undergo laboratory testing for other reasons as part of their normal medical care. In absence of diagnosis, abnormal laboratory findings (e.g. clinical chemistry, haematology,) or other abnormal assessments (e.g. physical examination findings) that are judged by the investigator to be clinically significant will be recorded as AE or SAE if they meet the definition of an AE or SAE. Stable and or chronic health conditions that were present at baseline and significantly worsen following the start of the study will also be reported as AEs or SAEs. However, abnormal laboratory findings associated with existing health condition, unless judged by the investigator as more severe than expected for the participant’s condition, will not be reported as AEs or SAEs. The investigator will exercise his or her medical and scientific judgment in deciding whether an abnormal laboratory finding, or other abnormal assessment is clinically significant.

**7.2 Detection of adverse events**

All participants will be instructed on the recognition of symptoms and signs by the study staff. Monitoring for any signs or symptoms of COVID-19 is instrumental in evaluating the efficacy of

Page **62** of **84**

DocuSign Envelope ID: F44BE397-221A-4F0B-840B-52A8EAA64913 DocuSign Envelope ID: CD06F8BF-7BA1-4865-8AD6-CDF085D4307E

Protocol FH-58 (AD5NCOV-HIV2b) Version 1.5 September 10^th^, 2021

the candidate vaccine. Participants will be monitored for incidence of local and systemic AE, in addition to monitoring for SAE. These participants will be instructed on how to take their temperature orally, as well as to record any symptoms in their e-diary or diary card for 28 days after receiving the vaccination. A thermometer and access to an e-diary or a paper diary card will be provided to record solicited AEs from First Injection Visit-Day 7, and unsolicited AEs from First Injection Visit-Day 28. Participants will review their completed e-diary or diary card with study staff at their second in-person visit on Day 28. A second e-diary or a paper diary card will be provided at Day 56 and will be collected at Day 84. Data collected from e-diaries and diary cards will be uploaded into the participant’s eCRF. All participants will be monitored for the incidence of SAE and MAE up to 52 weeks after receiving the vaccination, via a phone call every 4 weeks. Participants will be instructed to contact study staff by phone (24 hours a day), if a MAE or SAE occurs.

**AE Type Solicited AEs/ Unsolicited AEs/MAEs/SAEs Method of ‘solicited’ participant contact** E-diary or diary card

**Method of ‘unsolicited’ participant contact** E-diary or diary card**

**Method for reporting SAEs/MAEs** SAE report forms

**Method for reporting pregnancies** Pregnancy report forms

**Time period for detecting and recording adverse events and serious adverse events**

All AEs starting within 28 days following administration of the dose of the study productmust be recorded into the appropriate section of the eCRF, irrespective of intensity or whether or not they are considered injection related.

The time period for collecting and recording SAEs and MAEs in all participants will begin at the first receipt of study product/comparator and will end on Day 364.

All AEs/SAEs leading to withdrawal from the study will be collected and recorded from the time of the first receipt of study product. SAEs that are related to the investigational product will be collected and recorded from the time of the first receipt of study product/comparator until the participant is discharged from the study.

In addition to the above-mentioned reporting requirements and in order to fulfill international reporting obligations, SAEs that are related to study participation (i.e. protocol-mandated procedures, invasive tests, a change from existing therapy) will be collected and recorded from the time the participant consents to participate in the study until she/he is discharged from the study.

Page **63** of **84**

DocuSign Envelope ID: F44BE397-221A-4F0B-840B-52A8EAA64913 DocuSign Envelope ID: CD06F8BF-7BA1-4865-8AD6-CDF085D4307E

Protocol FH-58 (AD5NCOV-HIV2b) Version 1.5 September 10^th^, 2021

**Post-Study adverse events and serious adverse events**

A post-study AE/SAE is defined as any event that occurs outside of the AE/SAE reporting period. Investigators are not obligated to actively seek AEs or SAEs in former study participants. However, if the investigator learns of any SAE at any time after a participant has been discharged from the study, and he/she considers the event reasonably related to the investigational product, the investigator will promptly notify the Study Contact for Reporting SAEs.

**Evaluation of adverse events and serious adverse events**

**Active questioning to detect adverse events and serious adverse events**

As a consistent method of collecting AEs, the participants should be asked a non-leading question such as:

‘*Have you acted differently or felt different in any way since receiving the product or since the last visit?*’

When an AE/SAE occurs, it is the responsibility of the investigator to review all documentation (e.g. hospital progress notes, laboratory and diagnostics reports) relative to the event. The investigator will then record all relevant information regarding an AE/SAE in the eCRF. The investigator will attempt to establish a diagnosis pertaining to the event based on signs, symptoms, and/or other clinical information. In such cases, the diagnosis should be documented as the AE/SAE and not the individual signs/symptoms.

**Assessment of adverse events**

**Assessment of intensity**

The intensity of the following solicited AEs will be assessed as described:

The maximum intensity of local injection site redness/swelling will be scored as follows: 0: ≤ 20 mm

1: > 20 - ≤ 50 mm

2: > 50 - ≤ 100 mm

3: > 100 mm

The grade of fever will be scored as follows (oral temperatures*):

1: ≥ 38.0°C (100.4°F) - ≤ 38.5°C (101.3°F)

2: > 38.5°C (101.3°F) - ≤ 39.0°C (102.2°F)

3: > 39.0°C (102.2°F) - ≤ 40.0°C (104°F)

4: > 40.0°C (104°F)

*oral temperature=axillary temperature+0.2°C

Page **64** of **84**

DocuSign Envelope ID: F44BE397-221A-4F0B-840B-52A8EAA64913 DocuSign Envelope ID: CD06F8BF-7BA1-4865-8AD6-CDF085D4307E

Protocol FH-58 (AD5NCOV-HIV2b) Version 1.5 September 10^th^, 2021

The investigator will assess the maximum intensity that occurred over the duration of the event for all unsolicited AEs (including SAEs) recorded during the study. The assessment will be based on the investigator’s clinical judgment.

The intensity should be assigned to one of the following categories:

● Grade 1 (mild) **=** An AE which is easily tolerated by the participant, causing minimal discomfort and not interfering with everyday activities.

● Grade 2 (moderate) **=** An AE which is sufficiently discomforting to interfere with normal everyday activities.

● Grade 3 (severe) **=** An AE which prevents normal, everyday activities

● Grade 4= An adverse event that is potentially life threatening.

● Grade 5=An AE which results in death.

An AE that is assessed as Grade 3 (severe) should not be confused with a SAE. Grade 3 is a category used for rating the intensity of an event; and both AEs and SAEs can be assessed as Grade 3. An event is defined as ‘serious’ when it meets one of the predefined outcomes.

**Assessment of causality**

The investigator is obligated to assess the relationship between investigational product and the occurrence of each AE/SAE. The investigator will use clinical judgment to determine the relationship. Alternative plausible causes, such as natural history of the underlying diseases, concomitant therapy, other risk factors, and the temporal relationship of the event to the investigational product will be considered and investigated. The investigator will also consult the IB and/or PI to determine his/her assessment.

There may be situations when a SAE has occurred, but the investigator has minimal information to include in the initial SAE report. However, it is very important that the investigator always assesses causality for every event prior to submission of the SAE report. The investigator may change his/her opinion of causality in light of participant contact information and update the SAE information accordingly. The causality assessment is one of the criteria used when determining regulatory reporting requirements.

In case of concomitant administration of multiple products, it may not be possible to determine the causal relationship of general AEs to the individual products administered. The investigator should, therefore, assess whether the AE could be causally related to injection rather than to the individual products.

All solicited local (injection site) reactions will be considered causally related to injection. Causality of all other AEs should be assessed by the investigator using the following question:

*Is there a reasonable possibility that the AE may have been caused by the investigational product?*

Page **65** of **84**

DocuSign Envelope ID: F44BE397-221A-4F0B-840B-52A8EAA64913 DocuSign Envelope ID: CD06F8BF-7BA1-4865-8AD6-CDF085D4307E

Protocol FH-58 (AD5NCOV-HIV2b) Version 1.5 September 10^th^, 2021

**YES:** There is a reasonable possibility that the product(s) contributed to the AE.

**NO:** There is no reasonable possibility that the AE is causally related to the administration of the study product(s). There are other, more likely causes and administration of the study product(s) is not suspected to have contributed to the AE.

If an event meets the criteria to be determined as ‘serious’, additional examinations/tests will be performed by the investigator in order to determine ALL possible contributing factors for each SAE.

Possible contributing factors include:

● Medical history.

● Other medication.

● Protocol-required procedure.

● Other procedure not required by the protocol.

● Lack of efficacy of the product, if applicable.

● Erroneous administration.

● Other cause (specify).

**Assessment of outcomes**

The investigator will assess the outcome of all unsolicited AEs (including SAEs) recorded during the study as:

● Recovered/resolved.

● Recovering/resolving.

● Not recovered/not resolved.

● Recovered with sequelae/resolved with sequelae.

● Fatal (SAEs only).

**Medically attended AEs (MAEs)**

For each solicited and unsolicited symptom the participant experiences, the participant will be asked if they received medical attention defined as hospitalisation, or an otherwise unscheduled visit to or from medical personnel for any reason, including emergency room visits. This information will be recorded in the in the eCRF.

**Prompt reporting of SAE, SUSAR and other events to IDMC, Research Ethics Board (IRB)**

SAEs will be reported promptly to the Independent Data Monitoring Committee and IRB within the timeframes described in this protocol once the investigator determines that the event meets the protocol definition of a SAE. SAEs will be collected throughout the study. If anSAE is confirmed as aSUSAR, then the sponsor will report it to the IDMC.

Page **66** of **84**

DocuSign Envelope ID: F44BE397-221A-4F0B-840B-52A8EAA64913 DocuSign Envelope ID: CD06F8BF-7BA1-4865-8AD6-CDF085D4307E

Protocol FH-58 (AD5NCOV-HIV2b) Version 1.5 September 10^th^, 2021

TABLE 4. TIMEFRAMES FOR SUBMITTING SAES TO RESEARCH ETHICS BOARD AND STUDY FARMACOVIGILANCE UNIT

**Type of Event**

**Initial Reports Participant contact of Relevant Information on a Previous Report**

**Timeframe Documents Timeframe Documents**

**SAEs** 24 hours* SAE report 24 hours* SAE report * Timeframe allowed after receipt or awareness of the information.

**Contact information for reporting serious adverse events and Back-up Study Contact for Reporting SAEs**

24/24 hour and 7/7-day availability:

Email: fvcansino@huesped.org.ar; uam@huesped.org.ar

Details and contact information will be described in a separate Pharmacovigilance Agreement. **Completion and transmission of SAE reports**

Once an investigator becomes aware that a SAE has occurred in a study participant, the investigator (or designate) must complete the information in the SAE report as thoroughly as possible with all available details of the event, WITHIN 24 HOURS. Even if the investigator does not have all information regarding a SAE, the report should still be completed within 24 hours. Once additional relevant information is received, the report should be updated WITHIN 24 HOURS. The investigator will always provide an assessment of causality at the time of the initial report.

**Updating of SAE information after freezing of the participant’s eCRF**

When additional SAE information is received after freezing of the participant’s eCRF, new or updated information should be recorded on a paper report, with all changes signed and dated by the investigator. The updated report is provided to the IRB.

**Regulatory reporting requirements for serious adverse events**

The investigator will promptly report all SAEs to the in accordance with the procedures detailed in this protocol. Fundación Huésped as the clinical trial sponsor, has a legal responsibility to promptly notify the appropriate health agency about the safety of a product under clinical investigation. Prompt notification of SAEs by the investigator to the Study Contact for Reporting SAEs is essential so that legal obligations and ethical responsibilities towards the safety of other

Page **67** of **84**

DocuSign Envelope ID: F44BE397-221A-4F0B-840B-52A8EAA64913 DocuSign Envelope ID: CD06F8BF-7BA1-4865-8AD6-CDF085D4307E

Protocol FH-58 (AD5NCOV-HIV2b) Version 1.5 September 10^th^, 2021

participants are met. The purpose of the report is to fulfill specific regulatory and GCP requirements, regarding the product under investigation. All of these notifications will occur within 24 hours of the Investigator becoming aware of the SAE.

**7.3 Participant contact of adverse events and serious adverse events**

**Participant contacts during the study**

After the initial AE/SAE report, the investigator is required to proactively follow each participant and provide additional relevant information on the participant’s condition to the IRB.

All SAEs documented at a previous visit/contact and designated as not recovered/not resolved or recovering/resolving will be reviewed at subsequent visits/contacts until the end of the study.

With the exception of MAEs, all AEs documented at a previous visit/contact and designated as not recovered/not resolved or recovering/resolving will be reviewed at subsequent visits/contacts until 28 days after vaccination.

**Participant contact after the participant is discharged from the study**

The investigator will follow participants:

● With SAEs, or participants withdrawn from the study as a result of an AE, until the event has resolved, subsided, stabilized, disappeared, or until the event is otherwise explained, or the participant is lost to participant contact, defined as four attempts to contact by phone/email at different times of the day have been unsuccessful and a letter has been sent to the address with no reply.

● With MAE until the end of the study or the participants are lost to participant contact. ● With other non-serious AEs, until Day 28 after injection or they are lost to participant contact.

If the investigator receives additional relevant information on a previously reported SAE, he/she will provide this information to the IRB using a paper SAE and/or pregnancy report as applicable.

The manufacturer of the investigational product may request that the investigator performs or arranges the conduct of additional clinical examinations/tests and/or evaluations to elucidate as fully as possible the nature and/or causality of the AE or SAE. The investigator is obliged to assist. If a participant dies during participation in the study or during a recognized participant contact period, the IRB will be provided with any available post-mortem findings, including histopathology.

Page **68** of **84**

DocuSign Envelope ID: F44BE397-221A-4F0B-840B-52A8EAA64913 DocuSign Envelope ID: CD06F8BF-7BA1-4865-8AD6-CDF085D4307E

Protocol FH-58 (AD5NCOV-HIV2b) Version 1.5 September 10^th^, 2021

**7.4 Treatment of adverse events**

Treatment of any AE is at the sole discretion of the investigator and according to current good medical practice. Any medications administered for the treatment of an AE should be recorded in the participant’s eCRF.

**Participant card**

Study participants must be provided with the telephone number of the main contact for information about the clinical trial. Participants must be instructed to keep participant cards in their possession at all times.

The investigator (or designate) must therefore provide a “participant card” to each participant. In an emergency situation this card serves to inform the responsible attending physician that the participant is in a clinical study and that relevant information may be obtained by contacting the investigator.

**8. PARTICIPANT COMPLETION AND WITHDRAWAL**

**8.1 Participant completion**

A participant who returns for the concluding visit/is available for the concluding contact foreseen in the protocol is considered to have completed the study.

**8.2 Participant withdrawal**

Participants who are withdrawn because of SAEs/AEs must be clearly distinguished from participants who are withdrawn for other reasons. Investigators will follow participants who are withdrawn as result of a SAE/AE until resolution of the event. Withdrawals will not be replaced, unless the withdrawal is prior to the first dose of study vaccine.

**Participant withdrawal from the study**

From an analysis perspective, a ‘withdrawal’ from the study refers to any participant who did not come back for the concluding visit/was not available for the concluding contact foreseen in the protocol.

All data collected until the date of withdrawal/last contact of the participant will be used for the analysis.

A participant is considered a ‘withdrawal’ from the study when no study procedure has occurred, no participant contact has been performed and no further information has been collected for this participant from the date of withdrawal/last contact.

Page **69** of **84**

DocuSign Envelope ID: F44BE397-221A-4F0B-840B-52A8EAA64913 DocuSign Envelope ID: CD06F8BF-7BA1-4865-8AD6-CDF085D4307E

Protocol FH-58 (AD5NCOV-HIV2b) Version 1.5 September 10^th^, 2021

Investigators will make an attempt to contact those participants who do not return for scheduled visits or participant contact.

Information relative to the withdrawal will be documented in the eCRF. The investigator will document whether the decision to withdraw a participant from the study was made by the participant, or by the investigator, as well as which of the following possible reasons was responsible for withdrawal:

● SAE

● Non-serious AE

● Protocol violation (specify)

● Consent withdrawal, not due to an AE*

● Moved from the study area.

● Lost to participant contact.

● Other (specify).

**In case a participant is withdrawn from the study because he/she has withdrawn*

*consent, the investigator will document the reason for withdrawal of consent, if specified by the participant, in the eCRF.*

Participants who are withdrawn from the study because of SAEs/AEs must be clearly distinguished from participants who are withdrawn for other reasons. Investigators will follow participants who are withdrawn from the study as result of a SAE/AE until resolution of the event.

**Participant withdrawal from investigational product**

A ‘withdrawal’ from the investigational product refers to any participant who does not receive the complete treatment, i.e. when no further planned dose is administered from the date of withdrawal. A participant withdrawn from the investigational product may not necessarily be withdrawn from the study as further study procedures or participant contact may be performed (safety or immunogenicity) if planned in the study protocol.

Information relative to premature discontinuation of the investigational product will be documented on the Product Administration screen of the eCRF. The investigator will document whether the decision to discontinue further injection/treatment was made by the participant, or by the investigator, as well as which of the following possible reasons was responsible for withdrawal:

**●** SAE

**●** Non-serious AE

**●** Other (specify)

Page **70** of **84**

DocuSign Envelope ID: F44BE397-221A-4F0B-840B-52A8EAA64913 DocuSign Envelope ID: CD06F8BF-7BA1-4865-8AD6-CDF085D4307E

Protocol FH-58 (AD5NCOV-HIV2b) Version 1.5 September 10^th^, 2021

**9. OUTCOME MEASURES AND STATISTICAL METHODS**

**9.1 Sample Size Estimation**

Since this is an open, non-comparative study that seeks to observe the impact of vaccination on the evolution of HIV disease, a sample size defined by statistical criteria is not established.

**9.2 Study Population and Analysis Sets**

**9.2.1 Study Population**

**Intent-to-Treat Cohort**

The intent-to-treat cohort consists of all participants who receive at least one study injection, regardless of their protocol adherence and continued participation in the study. Participants will be analyzed according to their randomized treatment. Participants who withdraw from the study will be included up to the date of their study withdrawal.

**Per-protocol Analysis Cohort**

The per-protocol analysis cohort for safety includes subjects in the intent-to-treat who receive the correct treatment, the correct number of doses, and who do not have a major protocol deviation. Detailed definition of this analysis set including definition of major protocol deviations will be described in the Statistical Analysis Plan. The per-protocol analysis cohort for immunogenicity includes subjects in the per-protocol analysis cohort for safety whose samples were drawn inside of protocol specified time windows.

**9.3 Statistical Analysis**

This purpose of this section is to describe various planned analyses in this study. Details of statistical methods will be included in the Statistical Analysis Plan, which will be finalized prior to the first interim analysis.

**9.3.1 Demographics and Baseline Characteristics**

Demographic and baseline characteristics will be summarized overall and then by treatment group using appropriate descriptive statistics. Continuous data will be summarized using the number of observations, mean, standard deviation, median, minimum and maximum. Categorical data will be summarized using frequency counts and percentages. 95% confidence intervals will be reported, but no statistical hypothesis testing will be conducted. Results will be presented for all participants.

**9.3.2 Efficacy Analysis**

Due to limited sample size, no formal efficacy hypothesis will be tested. A listing of baseline characteristics, treatment group and outcome for virologically confirmed (PCR positive) serologically confirmed COVID-19 cases will be produced. An exploratory efficacy analysis of

Page **71** of **84**

DocuSign Envelope ID: F44BE397-221A-4F0B-840B-52A8EAA64913 DocuSign Envelope ID: CD06F8BF-7BA1-4865-8AD6-CDF085D4307E

Protocol FH-58 (AD5NCOV-HIV2b) Version 1.5 September 10^th^, 2021

Ad5-nCoV in preventing asymptomatic disease of COVID-19 will also be conducted at the end of the study.

**9.3.3 Safety Analysis**

**Primary Safety Endpoint:**

● The incidence of solicited adverse reactions within 7 days after vaccination.

● The incidence of unsolicited adverse events within 28 days after vaccination.

● The incidence of detectable viral load 24 and 52 weeks after vaccination

● The incidence of serious adverse events (SAE) and medically attended adverse events (MAE) within 52 weeks after vaccination in all participants.

**Secondary Safety Objective Endpoints**

● The incidence of a decrease in CD4+ cell count by ≥20% at 24 and 52 weeks after vaccination

● The delta in the CD4/CD8 rate compared to the basal value.

All SAEs will be listed and summarized by groups.

For each participant, the individual local events will be aggregated into a combined event “Local”, which is the maximum severity of the individual local events. For each participant, the individual general events will be aggregated into a combined event “General”, which is the maximum severity of the individual general events. An aggregate event “Any” will be defined as the maximal severity of the combined events “Local” and “General”. In addition to being graded for severity as mild, moderate or severe, the severity of all events will be graded as "Any", which will include mild, moderate or severe events, and "Significant" which will include moderate or severe events.

All statistical tests on safety performed will be 2-sided with Type I error of 5%. Missing values will not be included in the safety analyses, and there will be no imputation of missing values. No adjustments will be made for multiple comparisons.

For the analysis of proportions, binomial point estimates and exact binomial confidence intervals will be calculated.

Fisher’s Exact Test will be used to assess differences in rates of adverse events between treatment and control groups.

In addition, all safety data will be analysed descriptively, including unsolicited events collected through the end of the observation period.

The Safety analysis will be performed based on the intent-to-treat cohort and per-protocol cohort.

Page **72** of **84**

DocuSign Envelope ID: F44BE397-221A-4F0B-840B-52A8EAA64913 DocuSign Envelope ID: CD06F8BF-7BA1-4865-8AD6-CDF085D4307E

Protocol FH-58 (AD5NCOV-HIV2b) Version 1.5 September 10^th^, 2021

**9.3.4 Immunogenicity Analysis**

**Primary Immunogenicity Endpoints**:

● Evaluate the seroconversion rate of S-RBD IgG antibody on Day 28, Day 84, and Week 24 and Week 52 after vaccination, measured by ELISA.

● Evaluate the above-mentioned parameters comparing one versus two Ad5-nCoV doses. **Secondary Immunogenicity Endpoints**:

● GMT of S-RBD IgG antibody on Day 28, Day 84 and Week 24 and Week 52 after vaccination, measured by ELISA.

● GMI of S-RBD IgG antibody on Day 28, Day 84 and Week 24 and Week 52 after vaccination, measured by ELISA.

● Seroconversion rate of pseudo-virus neutralizing antibody on Day 28, Day 84 and Week 24 and Week 52 after vaccination.

● GMT of pseudo-virus neutralizing antibody on Day 28, Day 84 and Week 24 and Week 52 after vaccination.

● GMI of pseudo-virus neutralizing antibody on Day 28, Day 84 and Week 24 and Week 52 after vaccination.

● Positive rate and level of IL-2, IL-4, IL-13, and IFN-γ stimulated by peptide pool of S protein on days 28, Day 84 and Week 24 and Week 52 after vaccination, measured by intracellular cytokine staining (ICS).

Geometric mean antibody titers (GMTs) and GMI’s and their two side 95% confidence intervals will be calculated by group. Analyses will be performed on the logarithmically (base 10) transformed values. Individual titers below the detection limit will be set to half the limit.

For the analysis of proportions, binomial point estimates and exact binomial confidence intervals will be calculated for each group. Rates will be compared between groups using Fisher’s exact tests, and geometric means will be compared using t-tests.

All statistical tests performed for immunogenicity will be 2-sided with Type I error rate of 5%.

Missing values will not be included in the immunogenicity analyses, and there will be no imputation of missing values. No adjustments will be made for multiple comparisons.

Both per-protocol and intent-to-treat immunogenicity analyses will be carried out. The per protocol immunogenicity analysis set will exclude subjects whose samples were drawn outside of protocol specified time windows.

**9.3.5 Analysis of exploratory endpoints**

**Exploratory endpoints**

Page **73** of **84**

DocuSign Envelope ID: F44BE397-221A-4F0B-840B-52A8EAA64913 DocuSign Envelope ID: CD06F8BF-7BA1-4865-8AD6-CDF085D4307E

Protocol FH-58 (AD5NCOV-HIV2b) Version 1.5 September 10^th^, 2021

● Efficacy of two doses of Ad5-nCoV in preventing symptomatic disease of COVID-19 (confirmed by PCR) occurring 14 and 28 days to 52 weeks after vaccination, regardless of severity.

● Efficacy of two doses of Ad5-nCoV in preventing virologically (PCR) or serologically (four-fold increase in SARS-CoV-2 anti-N IgG from pre-immunization to post symptom, defined as Day 21-28 post illness blood test, or pre-symptom to post-symptom blood test) confirmed COVID-19 disease occurring 14 and 28 days to 52 weeks after vaccination, regardless of severity.

● Efficacy of two doses of Ad5-nCoV in preventing severe COVID-19 disease caused by SARS-CoV-2 infection from 14 and 28 days to 24 and 52 weeks after vaccination. ● Efficacy of two doses of Ad5-nCoV in preventing asymptomatic disease of COVID-19 (confirmed by N IgG antibody on week 52 after vaccination).

● Severity of COVID-19 cases among vaccine recipients (based on WHO or FDA criteria) as compared to the control group, to measure antibody-mediated disease enhancement (ADE).

● SARS-CoV-2 virus shedding in COVID-19 cases that occurred 28 days to 52 weeks after vaccination (detection of viral nucleic acid every 2 days after being confirmed).

● Genotype of SARS-CoV-2 virus isolates of COVID-19 cases that occurred 28 days to 52 weeks after vaccination.

● Suspected but unconfirmed cases of COVID-19 (either because of negative or no tests). ● Evaluate incidence of suspected but unconfirmed cases of COVID-19 (either because of negative or no tests).

Analysis of endpoints will be descriptive. PCR confirmed positive subjects will be classified as having severe or non-severe disease. Severity of disease, SARS-CoV-2 virus shedding and Genotypes SARS-CoV-2 virus isolates will be listed by subject and date, along with demographic data, including the study centre. Suspected but unconfirmed cases and asymptomatic cases will also be listed.

**10. DATA MANAGEMENT**

**10.1 General Requirements**

The data management of this clinical trial will follow the **CDISC** standard. A designated person (Central Data Manager) will be responsible for the data management of global clinical trial and develop a core data management plan approved by the global PI and the sponsor. The local PI of each participating country along with the Sponsor will identify a local CRO to monitor data entry and ensure compliance with the CDISC standard.

Page **74** of **84**

DocuSign Envelope ID: F44BE397-221A-4F0B-840B-52A8EAA64913 DocuSign Envelope ID: CD06F8BF-7BA1-4865-8AD6-CDF085D4307E

Protocol FH-58 (AD5NCOV-HIV2b) Version 1.5 September 10^th^, 2021

**10.2 Electronic Data Capture System**

This clinical trial will use a single, designated Electronic Data Capture System (EDC) in all participating countries. The local PI of each country must ensure that its research team is well trained in EDC system operation skills.

**11. STUDY SUSPENSION CRITERIA AND SAFETY MONITORING**

The study in its entirety may be terminated prematurely with reasonable justification by Study Steering Committee, National Regulatory Authority (ANMAT), or the Ethics Committee with oversight responsibilities at any time, and/or individual participants may terminate their participation prematurely, or have their participation be terminated by the Investigator. An Independent Data Monitoring Committee (IDMC) will be established to review the safety data of every participant with a GRADE 4 adverse event. Meetings will also be held should a participant have an adverse event meeting a “holding rule”. The safety review will be conducted using blinded data. The efficacy review will use unblinded data, but interim reports will not disclose the data at least that IDMC suggest changes in the protocol due to efficacy concerns.

**11.1 Study Suspension Criteria**

If a suspension criterion is activated, the study will be put on hold, and further injections will not be administered until a safety review has been conducted. Should a suspension criterion be activated, the local PI will inform the global PI, Sponsors, and NRA within 24 hours.

Suspension criteria include:

● More than 15% of participants experience a Grade 3 AE beginning within 3 days after study injection (day of injection and 2 subsequent days) and persisting at Grade ≥ 3 on three consecutive days depending upon symptom severity and kinetics.

● A suspected, unexpected serious adverse reaction (SUSAR) occurs that is life-threatening or results in death.

● The IDMC assessed the potential safety risks in clinical studies.

● The IDMC assessed that the vaccine candidate might be ineffective.

**11.2 Study Early Termination Criteria**

● Required by the sponsor, or

● Required by the regulatory authority, or

● Required by an institutional review board (IRB)

**11.3 Process of Suspension of Injection and/ or Study Modification**

In the event that a safety signal is observed, the IDMC might decide to cancel injection of all groups (early termination of study) or selected groups.

In this case, for impacted groups:

Page **75** of **84**

DocuSign Envelope ID: F44BE397-221A-4F0B-840B-52A8EAA64913 DocuSign Envelope ID: CD06F8BF-7BA1-4865-8AD6-CDF085D4307E

Protocol FH-58 (AD5NCOV-HIV2b) Version 1.5 September 10^th^, 2021

**●** Participants who are already injected will continue all visits as planned.

**●** Participants who signed an informed consent but have not received any study product will be informed that their study participation will be stopped.

Details of the IDMC functioning are described in the IDMC Charter.

**12. STUDY GOVERNANCE**

The study will be managed by Fundación Huésped. It is Fundación Huésped responsibility to oversee all study site activities. There will also be a designated IDMC to continuously monitor and review data from safety and efficacy trial.

**12.1 Study Steering Committee**

A Study Steering Committee (SC) will be established by the sponsor as the management decision-making body for this clinical trial. The SC will provide guidance to Fundación Huésped who will be responsible to ensure that the conduct of the trial in each site is harmonized with respect to important aspects such as data collection, laboratory tests, and implementation of vaccination. Approval, revision, adaptive adjustment of the clinical trial protocol, and major issues such as suspension and termination of clinical trials will all be decided by SC.

The composition of SC is as follows:

● Representatives from the sponsor

● PI and co-Global co-PI

● Central Safety Physician

● Central Statistician

**The main responsibilities of PI and Global co-PI are:**

● Assisting the sponsor to formulate the clinical trial protocol.

● In the course of clinical trials, coordinating the progress of each clinical trial site, and making recommendations for adaptive adjustment according to changes in circumstances (e.g., COVID-19 attack rate).

● When necessary, working with country PI to resolve issues that arise in the course of clinical trials.

● Preparing study reports for clinical trial.

● Working with the Study Statistician to finalize the Statistical Analysis Plan (SAP)

● Drafting local implementation documents and informed consent form (ICF) based on the clinical trial protocol and ICF.

Page **76** of **84**

DocuSign Envelope ID: F44BE397-221A-4F0B-840B-52A8EAA64913 DocuSign Envelope ID: CD06F8BF-7BA1-4865-8AD6-CDF085D4307E

Protocol FH-58 (AD5NCOV-HIV2b) Version 1.5 September 10^th^, 2021

● Submission of protocol, ICF, and other study documentation to the local IRB.

● Conducting clinical trials based on the approved implementation protocol.

● Solving the problems that occurred during the clinical trial.

● Preparing study reports for the sub-center clinical trial.

**12.2 Endpoint Review Committee**

The Endpoint Review Committee (ERC) will be blinded to the treatment assignments. Each endpoint case will be reviewed independently by two ERC members to confirm endpoint and severity; if their adjudications conflict, the endpoint will also be reviewed by a third member and the endpoint will be adjudicated by the majority vote. Members of the ERC will be appointed by the Study Steering Committee. Each positive laboratory result will be reviewed by the ERC. A detailed outline of ERC regulations and endpoint case criteria, in addition to the criteria defined below, will be available in the ERC charter.

Endpoints cases are defined by any of the following events:

1. A participant has an illness visit and virological confirmation (positive PCR test). PCR SARS-CoV-2 tests are permitted to be carried out at local study sites. Every effort will be made to verify positive PCR tests in a central reference laboratory.

2. A participant has an illness visit and serological confirmation of SARS-CoV-2. A positive serology test is defined by a four-fold rise or greater to N antibody titers in association with the illness.

3. A participant has illness visit and both virological and serological confirmation. 4. A participant who does not present with any symptoms (does not have any illness visits) but demonstrates seroconversion to the N antigen between the Injection and Week 52 visits.

**12.3 Independent Data Monitoring Committee**

The sponsor will establish an Independent Data Monitoring Committee (IDMC) consisting of at least 3 members to review global clinical trial safety and efficacy data. The IDMC core members will include an independent safety physician, clinical trial experts, an independent expert physician, infectious disease clinicians, biostatisticians, and epidemiologists who are not otherwise involved in the conduct of the project.

IDMC’s main responsibilities are:

● Reviewing the reported safety data in the participants after vaccination.

● Assessing the safety, if necessary, in the context of efficacy, of the investigational vaccine to safeguard the interests of trial participants.

Page **77** of **84**

DocuSign Envelope ID: F44BE397-221A-4F0B-840B-52A8EAA64913 DocuSign Envelope ID: CD06F8BF-7BA1-4865-8AD6-CDF085D4307E

Protocol FH-58 (AD5NCOV-HIV2b) Version 1.5 September 10^th^, 2021

● Conducting interim analysis and terminate the trial early (for benefit or futility) when sufficient evidence of efficacy is obtained.

● Monitoring the overall conduct of the clinical trial to protect its validity and credibility.

**13. ETHICAL AND REGULATORY CONSIDERATIONS**

This study will be conducted in accordance with all regulatory requirements. The trial will be conducted in accordance with the latest version of the Declaration of Helsinki, GCP, ICH regulatory guidelines, and requirements regarding ethical committee review, informed consent, and other statutes and regulations regarding the protection of the rights and wellbeing of participants in the study.

Each enrolment site and local investigator will be responsible for submitting this protocol, the informed consent document, and all recruiting materials to their local independent Research Ethics Board (IRB) for review and approval.

No changes will be made to the protocol without IRB approval, except where necessary to eliminate apparent immediate hazards to participants.

Participants will be able to withdraw their consents to participate at any time without giving a reason and with assurance that their care will not be affected in any way. The investigators may withdraw a participant if, in the investigator’s clinical judgment, it is in the best interest of the participant or if the participant is unable to comply with study requirements.

**13.1 Benefits/Potential Risks**

A complete description of the potential risks and benefits can be found in the informed consent document and Investigator Brochure [10].

**13.2 Informed Consent Process and Documentation**

The investigator or designate person will be responsible for presenting a full description of the research project including risks/benefits, and how personal health information may be used and disclosed in research. A written informed consent/authorization will then be obtained from the

participant prior to the screening procedures and injection. The principal investigator or designate will also be responsible for maintaining up-to-date records of the consent forms and providing a copy to the participant.

Participants will be encouraged and will have ample opportunity to have their questions answered before and after consenting to participate.

**13.3 Modification of the Protocol**

No modifications of the protocol may be made by study personnel without consultation the principal investigator. Any protocol amendment will be submitted to the appropriate local regulatory authority and to the Research Ethics Board(s) for approval.

Page **78** of **84**

DocuSign Envelope ID: F44BE397-221A-4F0B-840B-52A8EAA64913 DocuSign Envelope ID: CD06F8BF-7BA1-4865-8AD6-CDF085D4307E

Protocol FH-58 (AD5NCOV-HIV2b) Version 1.5 September 10^th^, 2021

**13.4 Interruption of the Trial**

Study suspension and early termination are described in Section 12.

**13.5 Confidentiality of Data and Access to Participant Records**

Confidentiality will be maintained within legal limits in the review of medical records and consent forms, which may contain the identity of the participant. Such records will be coded only with the participant’s initials and study number.

**13.6 Monitoring**

The sponsor will assign a professional staff to monitor the clinical trial, following the implementation protocol. The sponsor has a clinical trial quality management system and experience in the management of vaccine clinical trials. Monitors will follow the research sites’ SOP for clinical trial monitoring.

The study monitoring will start once the first participant has been enrolled in each site, during the study at appropriate intervals, and after the last participant has completed the study. The monitoring visit schedule will be determined by the sponsor and principal investigator based on the monitoring plan, the frequency of enrolments and participant contact visits. A monitor visit tracking form and report will be required for each monitoring visit.

**13.7 Auditing**

Audits or inspections may be made by the IRB to ensure that the study has been conducted in accordance with the protocol, FDA, ICH/GCP, and the ANMAT. Inspections may also be performed by a Country’s regulatory health authority at any point in the study.

**13.8 Archiving**

The investigators will retain all generated documents and study records on site, including informed consent forms and copies of case report forms for 10 years after completion of the study.

**13.9 Stipends for Participation**

Participants will be provided a stipend according to local practice to compensate for their time and travel required for each visit to the study site.

**13.10 Adverse Event Compensation and Insurance**

In the occurrence of an adverse event, the participant will be evaluated and treated by the investigators in accordance with local regulations. An insurance contract will be issued by the sponsor before starting the trial.

Page **79** of **84**

DocuSign Envelope ID: F44BE397-221A-4F0B-840B-52A8EAA64913 DocuSign Envelope ID: CD06F8BF-7BA1-4865-8AD6-CDF085D4307E

Protocol FH-58 (AD5NCOV-HIV2b) Version 1.5 September 10^th^, 2021

**13.11 Publication Policy**

Study result manuscripts will be prepared by the Principal Investigator and Global co-PI, reviewed by all investigators and the study sponsor, and submitted to peer-reviewed journals and scientific meetings within 24 months of the last participant contact.

**14. ADMINISTRATIVE MATTERS**

To comply with ICH GCP administrative obligations relating to data collection, monitoring, archiving data, audits, confidentiality and publications must be fulfilled.

**14.1 Data Entry Instructions**

Clinical data management will be performed at each enrollment site, following SOP standards and data cleaning procedures and under the supervision of the appointed Data Manager. Data entry will be performed by each of the study sites.

Completed eCRFs are reviewed by the external Monitor at the study site. When omissions or inconsistencies are detected by eCRF review, clarification or correction of the eCRF may be necessary, following approval by the investigator or appropriately qualified designee. In all cases, the investigator remains accountable for the study data.

**14.2 Monitoring**

It is the responsibility of monitors to:

● Verify that all participants have been enrolled according to the protocol and that informed consent has been obtained prior to any study procedure.

● Verify that data is authentic, accurate, and complete.

● Review all eCRFs after data entry

● Verify that study files, the participant files, source documents, eCRFs, any SAE forms, product logs, and laboratory records are completed properly to ensure that the study is being conducted according to the protocol and GCP.

● Safety and rights of participants are being protected throughout the study.

● Study is conducted in accordance with the currently approved protocol, any other study agreements, GCP and all applicable regulatory requirements.

Direct access to all study-site related and source data is mandatory for the purpose of monitoring review. The investigators and the head of the medical institutions (where applicable) agrees to allow the monitor direct access to all relevant documents.

The investigators must ensure provision of reasonable time, space and qualified personnel for monitoring visits.

Page **80** of **84**
